# The endothelial activation and stress index as a predictor of 28-day mortality in pulmonary sepsis: a retrospective two-cohort analysis

**DOI:** 10.3389/fmed.2026.1714682

**Published:** 2026-01-27

**Authors:** Chunxia Yang, Shiting Zhou, Chunyan Wen, Jun Chen

**Affiliations:** Department of Respiratory and Critical Care Medicine, Zhejiang Provincial People's Hospital Bijie Hospital, Bijie, China

**Keywords:** endothelial activation and stress index, intensive care, pneumonia, pulmonary sepsis, risk stratification

## Abstract

**Background:**

The Endothelial Activation and Stress Index (EASIX) is an emerging biomarker that serves as a straightforward and objective measure of systemic endothelial dysfunction and critical illness severity. This study aims to evaluate the prognostic value of EASIX for 28-day mortality in patients with pulmonary sepsis.

**Materials and methods:**

This retrospective study utilised a two-cohort design. The internal cohort was derived from MIMIC-IV; an external cohort was derived from a tertiary hospital (2022–2025). The association between the EASIX and 28-day mortality was evaluated using multivariable Cox regression, restricted cubic spline (RCS) analysis, and Kaplan–Meier survival curves. An ensemble machine-learning approach (Boruta, LASSO-COX, XGBoost, and SVM) was employed for feature selection. Significant predictors were incorporated into a multivariate Cox model to construct a prognostic nomogram. The model’s discriminative performance was assessed using receiver operating characteristic (ROC) curves and the area under the curve (AUC), and compared against conventional severity scores.

**Results:**

A total of 5,416 patients were analyzed. In multivariable adjusted models, the EASIX emerged as an independent predictor of short term mortality. Each unit increase in EASIX was associated with a 7% higher risk of 28-day ICU death (HR 1.07, 95% CI 1.05–1.11, *p* < 0.001). A clear dose–response relationship was observed across EASIX quartiles, with mortality rising from 13.29% (Q1) to 27.92% (Q4); patients in Q4 had nearly twice the mortality risk of those in Q1 (HR 1.99, 95% CI 1.60–2.46). RCS analysis revealed a nonlinear relationship. Machine-learning feature selection consistently identified EASIX as a core variable. The final prognostic model, integrating EASIX with five other clinical features, demonstrated stable and superior discriminative ability (AUC 0.67–0.73) compared to traditional severity scores in both internal and external validation.

**Conclusion:**

EASIX is a potent and independent predictor of short-term mortality in pulmonary sepsis. Its integration into a pragmatic prognostic model enhances early risk stratification, highlighting its potential as a readily available clinical tool.

## Introduction

Pulmonary sepsis, defined as sepsis triggered by a primary pulmonary infection, represents one of the most common and characteristic forms of sepsis encountered in clinical practice (1–3). According to World Health Organisation (WHO) epidemiological data, approximately 10–30% of patients with community-acquired pneumonia (CAP) develop sepsis, a proportion that rises to nearly 50% among those severe enough to require intensive care. Hospital-acquired pneumonia (HAP/VAP) represents another major cause, particularly in mechanically ventilated patients. Mortality from pulmonary sepsis remains alarmingly high, ranging from 30 to 50%, and exceeds 50% once septic shock supervenes (4–7). These persistently high mortality rates pose substantial challenges to effective patient management. Hence, the development of accurate and clinically applicable prognostic models is essential to guide medical decision-making and potentially improve outcomes in this high-risk population.

The Endothelial Activation and Stress Index (EASIX) is an emerging and powerful biomarker. Its primary value lies in its ability to non-invasively, simply, and quantitatively assess the degree of endothelial cell activation and damage *in vivo* (8). EASIX is calculated using lactate dehydrogenase (LDH), serum creatinine, and platelet count (PLT)—each component reflecting a distinct aspect of endothelial injury and dysfunction. LDH, a marker of cellular injury and death, increases in response to the lysis of endothelial and other tissue cells during a cytokine storm. Creatinine, an indicator of renal function, rises as kidney function declines—a direct consequence of endothelial injury causing microvascular ischaemia and thrombotic microangiopathy (TMA), given the kidney’s dense capillary network (9–12). A decrease in platelet count reflects consumptive loss due to increased expression of procoagulant factors on activated endothelial surfaces, which recruits platelets and promotes microthrombus formation (13). Endothelial activation is central to sepsis pathogenesis. While initial endothelial responses facilitate immune cell trafficking (14–16), dysregulated activation leads to widespread vascular leakage, contributing to acute respiratory distress syndrome (ARDS)—a major cause of death in pulmonary sepsis (17, 18). EASIX thus translates this complex endothelial pathophysiology into a readily calculable clinical tool, potentially enabling early identification of high-risk patients and guiding timely intervention.

EASIX was originally developed and extensively validated in haematology, particularly among patients undergoing allogeneic haematopoietic stem cell transplantation (allo-HSCT) (19). With growing research, the potential applications of EASIX have expanded well beyond transplantation. It has demonstrated unique and significant prognostic utility in various critical illnesses, including cardiovascular and cerebrovascular diseases, as well as severe asthma (20, 21). However, as an emerging biomarker, its prognostic value in high-risk patients with pulmonary sepsis is still unclear. To address this knowledge gap, we conducted a retrospective cohort study using the large-scale Medical Information Mart for Intensive Care IV (MIMIC-IV) database as an internal cohort, supplemented with an external validation cohort. This study aims to comprehensively evaluate the relationship between EASIX and short-term outcomes in pulmonary sepsis patients and to provide clinically actionable strategies to improve risk stratification and personalised management in this vulnerable population.

## Materials and methods

### Data source

This study employed a retrospective two-cohort design to enhance the robustness and generalisability of the findings. The primary analysis was conducted using the Medical Information Mart for Intensive Care IV (MIMIC-IV) database, which contains comprehensive and de-identified clinical data from over 190,000 adult ICU patients admitted to a large U.S. academic medical centre between 2008 and 2019. This dataset served as the internal training cohort (22). To further evaluate the cross-institutional and cross-population applicability of the results, an external validation cohort was established, comprising patients with pulmonary sepsis admitted to the Department of Pulmonary and Critical Care Medicine at Bijie Hospital of Zhejiang Provincial People’s Hospital between June 2023 and June 2025. The study protocol was approved by the Ethics Committee of Bijie Hospital of Zhejiang Provincial People’s Hospital. The committee waived the requirement for individual informed consent due to the retrospective nature of the study and the use of fully anonymised data.

### Study population

**Figure 1:**
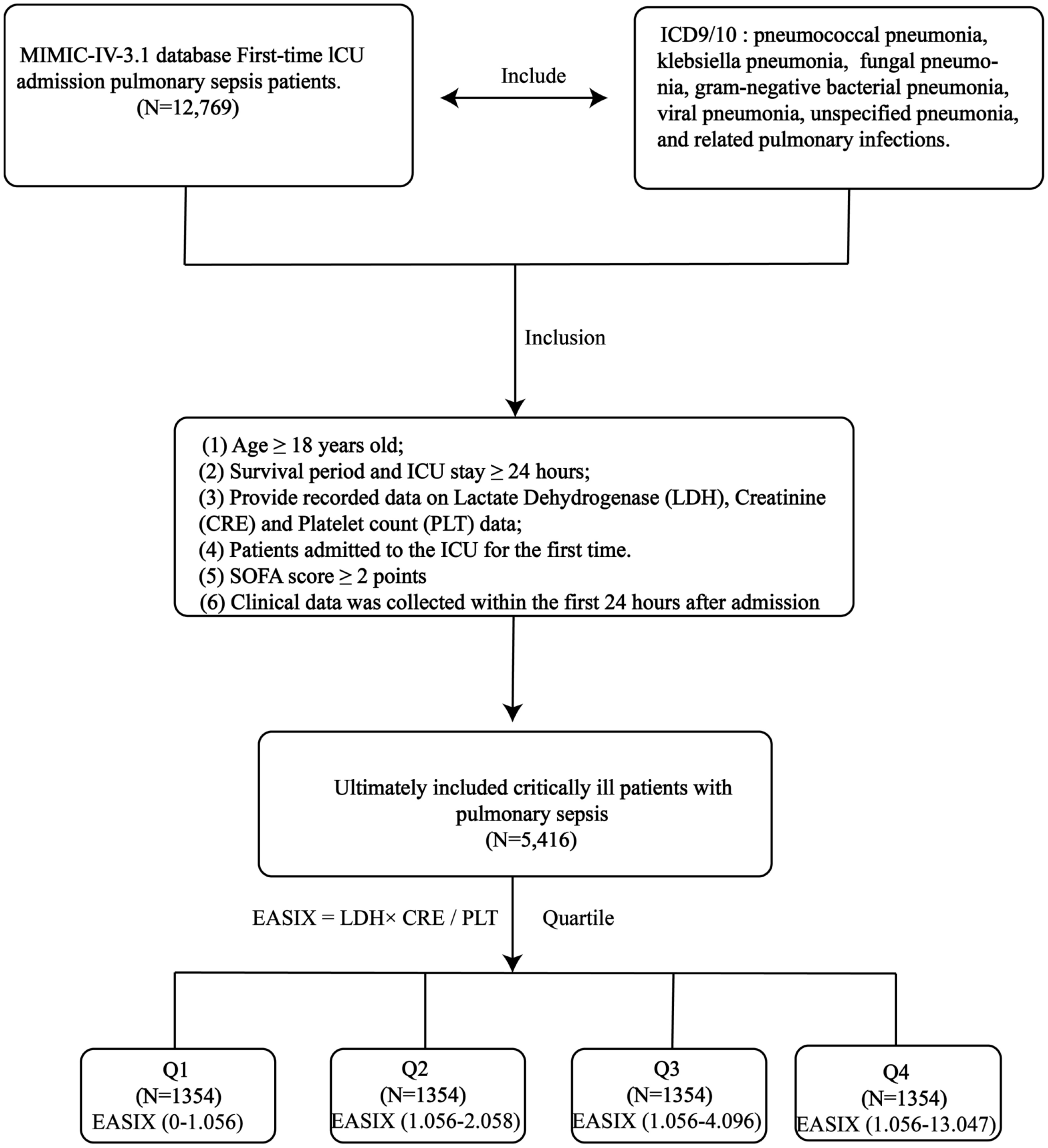
The research design and workflow of this study.

This study focused on patients with pulmonary sepsis who were admitted to the Intensive Care Unit (ICU) for the first time. Cases were identified using the International Classification of Diseases, Ninth and Tenth Revision (ICD-9/10) codes, including pneumococcal pneumonia, *Klebsiella pneumoniae* pneumonia, fungal pneumonia, viral pneumonia, Gram-negative bacterial pneumonia, aspiration pneumonia, unspecified pneumonia, and other related pulmonary infections. The inclusion criteria were as follows:(1) age ≥18 years; (2) survival and ICU stay both lasting ≥24 h; (3) availability of complete records for lactate dehydrogenase (LDH), serum creatinine (CRE), and platelet (PLT) levels; (4) first-time ICU admission; and (5) a Sequential Organ Failure Assessment (SOFA) score ≥2 at admission. Clinical data were collected within the first 24 h following ICU admission. Based on their EASIX (Endothelial Activation and Stress Index) scores, all patients were stratified into four groups (Q1–Q4) using quartile division (Figure 1).

### Variable extraction

Data extraction was performed using PostgreSQL (version 13.7.2) and Navicat Premium (version 16) by executing structured query language (SQL). The extracted variables were categorised into six groups:(1) Demographic data: age, sex, weight, height, and ethnicity; (2) Comorbidities: hypertension, acute kidney injury (AKI), chronic kidney disease (CKD), heart failure (HF), diabetes, hyperlipidaemia (HLD), chronic bronchitis (CB), ischaemic heart disease (IHD), and chronic obstructive pulmonary disease (COPD); (3) Vital signs: respiratory rate (RR), heart rate (HR), non-invasive systolic blood pressure (NISBP), non-invasive diastolic blood pressure (NIDBP), and oxygen saturation (SpO₂); (4) Disease severity scores at admission: Acute Physiology Score III (APS III), Simplified Acute Physiology Score II (SAPS II), Oxford Acute Severity of Illness Score (OASIS), Sequential Organ Failure Assessment (SOFA), Systemic Inflammatory Response Syndrome (SIRS) score, and Acute Physiology and Chronic Health Evaluation II (APACHE II); (5) Laboratory measurements: red blood cell (RBC), white blood cell (WBC), neutrophil count, hemoglobin (Hb), platelet count (PLT), red cell distribution width (RDW), hematocrit (HCT), albumin (ALB), anion gap (AG), lactate (Lac), serum sodium (Na), serum calcium (Ca), serum potassium (K), serum magnesium (Mg), serum chloride (Cl), glucose (GLU), total carbon dioxide (TCO₂), partial pressure of oxygen (pO₂), partial pressure of carbon dioxide (pCO₂), uric acid (UA), lactate dehydrogenase (LDH), pH, serum creatinine (CRE), blood urea nitrogen (BUN), international normalized ratio (INR), prothrombin time (PT), activated partial thromboplastin time (APTT), alanine aminotransferase (ALT), aspartate aminotransferase (AST), and total bilirubin (TB); (6) Treatment interventions: mechanical ventilation (MV), continuous renal replacement therapy (CRRT), antibiotic administration (ABX), vasopressor use (VP), glucocorticoid therapy (GC), and sedative/analgesic agents (Sa). To minimise bias, variables with a missing rate exceeding 30% were excluded from the analysis. For variables with less than 30% missing data, multiple imputation was performed using the “mice” package in R.

### Definition of EASIX and endpoints

The EASIX score was calculated using the following formula: EASIX = [Lactate Dehydrogenase (LDH, U/L) × Creatinine (CRE, mg/dL)] / Platelet count (PLT, ×10^9^/L) (8). The primary endpoint was 28-day ICU mortality, and the secondary endpoint was 28-day in-hospital all-cause mortality.

### Subgroup analysis

Subgroup analyses were performed according to pre-specified criteria, including age (>65 years vs. ≤65 years), sex, ethnicity, and the following comorbidities: hypertension, acute kidney injury (AKI), chronic kidney disease (CKD), heart failure (HF), diabetes, hyperlipidaemia (HLD), myocardial infarction (MI), chronic bronchitis (CB), ischaemic heart disease (IHD), and chronic obstructive pulmonary disease (COPD). Within each subgroup, a Cox proportional hazards regression model was applied for assessment, and forest plots were used to visually present the hazard ratios (HRs) along with their 95% confidence intervals (CIs).

### Feature selection, risk prediction modeling, and validation

Trend testing and variance inflation factor (VIF) were used to evaluate multicollinearity for the variables included in Modle3. Variables with a VIF exceeding 5 were removed (Supplementary Table 5). Within the internal cohort, all patients were randomly split into a test set and a training set at a ratio of 7:3. To identify the most robust predictors, we employed a multi-step, machine learning-informed feature selection strategy within the test set. First, we used the Boruta algorithm, a wrapper method built on Random Forest, to distinguish truly important features from noise by comparing them with randomly permuted shadow features. This provided an initial ranking of all candidate variables (23). Second, to achieve a parsimonious model and mitigate overfitting, we applied LASSO (Least Absolute Shrinkage and Selection Operator) regression with 10-fold cross-validation to shrink coefficients of less relevant variables to zero. Finally, to ensure robustness and consensus, we examined variable importance rankings from three additional algorithms: XGBoost, Gradient Boosting, and Support Vector Machine (SVM). Variables consistently ranked high across these diverse methods were considered core predictors. This consensus approach ensured that our final variable selection was data-driven, reproducible, and not overly reliant on a single algorithm’s bias. By comparing the feature rankings and importance scores derived from these models, we systematically identified a consensus set of core variables strongly associated with the primary endpoint (28-day ICU mortality). Subsequently, multivariable Cox proportional hazards regression was applied to these core variables to identify those with independent prognostic significance. A prognostic prediction model was then constructed based on these variables and visually presented as a nomogram. The discriminative ability of the model was evaluated using Receiver Operating Characteristic (ROC) curves and the Area Under the Curve (AUC). Finally, to validate the stability and generalisability of the constructed model, its predictive performance was independently tested on the external validation cohort.

### Statistical analysis

Continuous variables were expressed as median with interquartile range (IQR) or median (minimum-maximum), and between-group comparisons were performed using the Mann–Whitney U test or Kruskal-Wallis test as appropriate. Categorical variables were presented as frequency and percentage, and group differences were compared using Pearson’s chi-square test or Fisher’s exact test. The association between EASIX and 28-day mortality was evaluated using univariable and multivariable Cox proportional hazards regression. Three hierarchical models were constructed: Model 1 (unadjusted), Model 2 (adjusted for demographics and comorbidities), and Model 3 (fully adjusted for demographics, comorbidities, key interventions, and laboratory markers that differed significantly between survivors and non-survivors). The covariates included in Model 3 were selected based on clinical relevance and univariate significance; the final set comprised 34 variables, yielding an events-per-variable (EPV) ratio of 33.8 (1,114 events/33 variables), which substantially exceeds the recommended threshold of 10–20, thereby minimizing overfitting concerns. The proportional hazards assumption was assessed using Schoenfeld residuals and the corresponding global test. This evaluation was performed on the fully adjusted Model 3, which included EASIX as a continuous variable along with all other covariates (Supplementary Table S8). The assumption was tested for both the 28-day ICU mortality and 28-day in-hospital mortality models. In addition to the global test, we examined the assumption for the EASIX variable specifically. A visual assessment of the Schoenfeld residuals for EASIX is provided in Supplementary Figure S5. Restricted cubic splines with four knots (at the 5th, 35th, 65th, and 95th percentiles) were used to explore potential non-linear relationships. For model development, the internal cohort was randomly split into training and test sets (7:3). Feature selection was performed using the Boruta algorithm and LASSO-COX regression, followed by ensemble machine-learning methods (XGBoost, Gradient Boosting, SVM) to identify robust predictors. Model discrimination was evaluated using receiver operating characteristic (ROC) curves and the area under the curve (AUC). Statistical analyses were performed in R (version 4.2.2), with a two-sided *p*-value < 0.05 considered significant.

## Results

### Baseline characteristics of study participants

Based on the inclusion and exclusion criteria, a total of 5,416 patients with severe pulmonary sepsis from the MIMIC-IV database were included in the analysis. The 28-day ICU and in-hospital mortality rates were 20.57 and 19.07%, respectively. Table 1 summarises the detailed baseline characteristics of the patients stratified by EASIX quartiles. According to the EASIX index, patients were divided into four groups (Q1–Q4), with 1,354 patients in each group. Patients in higher EASIX quartiles were more likely to be male and had a greater burden of comorbidities (e.g., hypertension, AKI, CKD, diabetes, HF, MI, IHD). Disease severity scores (SOFA, APS III, SAPS II, Charlson index, APACHE II) increased significantly across ascending quartiles. Laboratory profiles also showed graded deterioration, with higher quartiles associated with lower hemoglobin and platelet counts, and elevated creatinine, urea, lactate, liver enzymes, bilirubin, RDW, and coagulation parameters (INR, PT, APTT). The use of intensive therapies (CRRT, vasopressors) rose progressively with EASIX. Critically, both ICU and in-hospital mortality demonstrated a clear dose–response relationship, increasing significantly across quartiles (*p* < 0.001) (Supplementary Tables S1, S2).

**Table 1:** Summary descriptives table by groups of EASIX group.

Variables	All	Q1	Q2	Q3	Q4	*P*-value
	*N* = 5,416	*N* = 1,354	*N* = 1,354	*N* = 1,354	*N* = 1,354	
EASIX	2.058 (0–13.047)	0.649 (0–1.056)	1.502 (1.056–2.058)	2.884 (2.058–4.096)	6.359 (4.096–13.047)	<0.001
Age	69 (18–104)	68 (18–104)	70 (19–99)	70 (20–98)	68 (19–97)	0.014
Gender						<0.001
F	2,211 (40.82%)	671 (49.56%)	563 (41.58%)	498 (36.78%)	479 (35.38%)	
M	3,205 (59.18%)	683 (50.44%)	791 (58.42%)	856 (63.22%)	875 (64.62%)	
Race						0.006
Other race	1984 (36.63%)	460 (33.97%)	520 (38.40%)	471 (34.79%)	533 (39.36%)	
White	3,432 (63.37%)	894 (66.03%)	834 (61.60%)	883 (65.21%)	821 (60.64%)	
Weight	78.9 (30–396.5)	74.45 (31.6–390.7)	78.2 (31–226)	81.25 (33.75–396.5)	81.2 (30–219.067)	<0.001
Complication						
Hypertension						<0.001
No	3,539 (65.34%)	774 (57.16%)	828 (61.15%)	934 (68.98%)	1,003 (74.08%)	
Yes	1877 (34.66%)	580 (42.84%)	526 (38.85%)	420 (31.02%)	351 (25.92%)	
AKI						<0.001
No	2,187 (40.38%)	879 (64.92%)	567 (41.88%)	430 (31.76%)	311 (22.97%)	
Yes	3,229 (59.62%)	475 (35.08%)	787 (58.12%)	924 (68.24%)	1,043 (77.03%)	
CKD						<0.001
No	4,094 (75.59%)	1,211 (89.44%)	1,086 (80.21%)	926 (68.39%)	871 (64.33%)	
Yes	1,322 (24.41%)	143 (10.56%)	268 (19.79%)	428 (31.61%)	483 (35.67%)	
Diabetes						<0.001
No	3,629 (67.01%)	1,006 (74.30%)	910 (67.21%)	871 (64.33%)	842 (62.19%)	
Yes	1787 (32.99%)	348 (25.70%)	444 (32.79%)	483 (35.67%)	512 (37.81%)	
HLD						<0.001
No	3,580 (66.10%)	954 (70.46%)	849 (62.70%)	868 (64.11%)	909 (67.13%)	
Yes	1836 (33.90%)	400 (29.54%)	505 (37.30%)	486 (35.89%)	445 (32.87%)	
CB						0.001
No	4,648 (85.82%)	1,140 (84.19%)	1,140 (84.19%)	1,166 (86.12%)	1,202 (88.77%)	
Yes	768 (14.18%)	214 (15.81%)	214 (15.81%)	188 (13.88%)	152 (11.23%)	
HF						<0.001
No	3,272 (60.41%)	940 (69.42%)	805 (59.45%)	768 (56.72%)	759 (56.06%)	
Yes	2,144 (39.59%)	414 (30.58%)	549 (40.55%)	586 (43.28%)	595 (43.94%)	
MI						<0.001
No	4,812 (88.85%)	1,288 (95.13%)	1,224 (90.40%)	1,182 (87.30%)	1,118 (82.57%)	
Yes	604 (11.15%)	66 (4.87%)	130 (9.60%)	172 (12.70%)	236 (17.43%)	
IHD						<0.001
No	3,440 (63.52%)	994 (73.41%)	865 (63.88%)	812 (59.97%)	769 (56.79%)	
Yes	1976 (36.48%)	360 (26.59%)	489 (36.12%)	542 (40.03%)	585 (43.21%)	
COPD						<0.001
No	4,238 (78.25%)	1,015 (74.96%)	1,042 (76.96%)	1,074 (79.32%)	1,107 (81.76%)	
Yes	1,178 (21.75%)	339 (25.04%)	312 (23.04%)	280 (20.68%)	247 (18.24%)	
SOFA	7 (2–21)	5 (2–16)	6 (2–17)	7 (2–20)	9 (2–21)	<0.001
APSIII	54 (7–178)	48 (9–139)	50 (7–142)	55 (16–149)	62 (16–178)	<0.001
SIRS	3 (0–4)	3 (0–4)	3 (0–4)	3 (0–4)	3 (0–4)	0.302
SAPSII	42 (6–106)	39 (8–91)	41 (6–94)	43 (6–106)	47 (10–102)	<0.001
OASIS	36 (9–67)	36 (11–61)	36 (12–61)	36 (9–67)	37 (12–64)	0.030
Charlson	6 (0–19)	5 (0–17)	6 (0–18)	6 (0–19)	6 (0–17)	<0.001
APACHEII	21 (1–52)	19 (3–40)	19 (2–45)	21 (1–46)	24 (5–52)	<0.001
HR	93 (0–191)	95 (0–191)	92 (37–189)	92 (0–182)	92 (0–179)	0.022
NBPS	118 (46–253)	119 (53–253)	120 (55–209)	118 (51–226)	116 (46–210)	0.003
NBPD	67 (12–6,868)	67.5 (12–199)	68 (14–144)	66 (22–159)	65 (15–6,868)	0.617
RR	20 (0–115)	20 (0–52)	20 (0–63)	20.5 (0–115)	21 (0–52)	0.721
Spo2	97 (36–963)	97 (46–100)	97 (36–100)	97 (58–100)	97 (54–963)	0.569
Temperature (°F)	98.3 (33.3–106)	98.4 (37.1–103.2)	98.4 (35.1–103.3)	98.3 (33.7–104.4)	98.2 (33.3–106)	0.030
HCT	32 (10.6–60.4)	32.1 (13.9–52.9)	33.1 (11.9–56)	31.9 (10.6–60.4)	30.25 (15–58.8)	<0.001
Hb	10.3 (3.6–19.6)	10.3 (4–17.4)	10.7 (3.6–17.5)	10.2 (3.6–19.6)	9.8 (4.6–19.4)	<0.001
PLT	197 (8–1,647)	281 (52–1,647)	207 (42–855)	173 (23–618)	140 (8–789)	<0.001
RDW	15.2 (11.1–33.1)	14.9 (11.4–25.9)	14.8 (11.1–29.3)	15.3 (11.6–33.1)	15.9 (11.8–32.1)	<0.001
RBC	3.46 (1.08–7.07)	3.52 (1.27–6.12)	3.61 (1.23–6.82)	3.45 (1.08–6.58)	3.26 (1.44–7.07)	<0.001
WBC	12.15 (0.1–344.4)	12.7 (0.3–63.8)	11.75 (0.3–156.2)	11.9 (0.1–344.4)	12.05 (0.1–267.2)	0.441
ALB	2.9 (0.5–5.5)	2.9 (0.6–5.1)	3 (1–4.9)	2.9 (1–4.8)	2.9 (0.5–5.5)	0.065
AG	15 (−4–89)	13 (1–89)	14 (3–45)	15 (1–47)	16 (−4–49)	<0.001
Ca	8.3 (1.5–16.8)	8.3 (1.5–12.4)	8.3 (1.9–12.1)	8.3 (4.4–12.6)	8.2 (4.2–16.8)	0.464
Cl	104 (61–153)	103 (61–130)	104 (67–135)	104 (70–136)	103 (64–153)	0.001
GLU	134 (10–2044)	128 (42–1,123)	132 (28–1,164)	135 (31–1,429)	139 (10–2044)	<0.001
K	4.2 (1.6–10)	4 (1.8–10)	4.1 (2.3–7.5)	4.2 (2.1–7.9)	4.4 (1.6–8.9)	<0.001
Na	139 (98–176)	139 (98–176)	139 (100–162)	139 (108–174)	138 (102–175)	<0.001
Mg	1.9 (0.3–7.5)	1.9 (0.3–5.9)	1.9 (0.6–7.5)	2 (0.7–6.5)	2 (0.9–7.2)	<0.001
TCO2	25 (0–61)	26 (7–61)	25 (5–56)	24 (4–56)	23 (0–59)	<0.001
Lac	1.7 (0.3–22)	1.4 (0.3–14)	1.7 (0.5–22)	1.8 (0.3–21)	2 (0.3–20.6)	<0.001
pco2	42 (10–232)	43 (15–232)	43 (10–148)	42.5 (13–148)	41 (16–116)	<0.001
ph	7.36 (6.56–7.7)	7.38 (6.88–7.64)	7.37 (6.56–7.64)	7.35 (6.84–7.65)	7.34 (6.95–7.7)	<0.001
po2	76 (14–681)	81 (18–544)	78 (14–649)	74 (17–550)	71 (15–681)	0.198
INR	1.3 (0.8–21.1)	1.3 (0.8–15.7)	1.3 (0.9–21.1)	1.4 (0.9–13.8)	1.4 (0.9–15)	<0.001
PT	14.6 (9–150)	14 (9–150)	14.3 (9.6–150)	14.9 (9.3–150)	15.7 (9.4–123.9)	<0.001
APTT	32 (17.1–150)	31 (17.8–150)	30.8 (17.1–150)	32.6 (19.2–150)	34.4 (19.9–150)	<0.001
ALT	26 (1–4,408)	21 (1–797)	24 (1–1,473)	29 (2–4,408)	34 (3–3,299)	<0.001
AST	39 (0–11,610)	26 (0–1,509)	36 (6–2,274)	45 (5–11,610)	60 (4–5,946)	<0.001
TB	0.6 (0–50.7)	0.5 (0–25.4)	0.6 (0.1–31.9)	0.7 (0.1–45.9)	0.8 (0.1–50.7)	<0.001
CRE	1.2 (0–15.1)	0.7 (0–3.4)	1.1 (0.3–7.4)	1.4 (0.3–10.8)	2 (0.3–15.1)	<0.001
BUN	25 (2–210)	17 (2–87)	23 (3–132)	29 (4–198)	39 (3–210)	<0.001
LDH	288 (60–11,300)	218 (60–825)	272 (87–1,345)	326 (104–3,153)	387 (103–11,300)	<0.001
Treatment						
Ventilation						0.857
No	397 (7.33%)	92 (6.79%)	101 (7.46%)	102 (7.53%)	102 (7.53%)	
Yes	5,019 (92.67%)	1,262 (93.21%)	1,253 (92.54%)	1,252 (92.47%)	1,252 (92.47%)	
CRRT						<0.001
No	4,888 (90.25)	1,327 (98.01)	1,290 (95.27)	1,211 (89.44)	1,060 (78.29%)	
Yes	528 (9.75)	27 (1.99)	64 (4.73)	143 (10.56)	294 (21.71%)	
Sa						0.582
No	1,237 (22.84%)	326 (24.08%)	296 (21.86%)	306 (22.60%)	309 (22.82%)	
Yes	4,179 (77.16%)	1,028 (75.92%)	1,058 (78.14%)	1,048 (77.40%)	1,045 (77.18%)	
GC						0.210
No	3,066 (56.61%)	775 (57.24%)	791 (58.42%)	762 (56.28%)	738 (54.51%)	
Yes	2,350 (43.39%)	579 (42.76%)	563 (41.58%)	592 (43.72%)	616 (45.49%)	
VP						<0.001
No	1,684 (31.09%)	492 (36.34%)	436 (32.20%)	391 (28.88%)	365 (26.96%)	
Yes	3,732 (68.91%)	862 (63.66%)	918 (67.80%)	963 (71.12%)	989 (73.04%)	
ABX						0.881
No	6 (0.11%)	2 (0.15%)	2 (0.15%)	1 (0.07%)	1 (0.07%)	
Yes	5,410 (99.89%)	1,352 (99.85%)	1,352 (99.85%)	1,353 (99.93%)	1,353 (99.93%)	
Hospital time	13.17 (0.08–248.45)	13.25 (0.08–181.69)	12.85 (0.47–174.58)	13.535 (0.24–190.03)	13.165 (0.26–248.45)	0.920
Hospital dead	1,033 (19.07%)	169 (12.48%)	230 (16.99%)	285 (21.05%)	349 (25.78%)	<0.001
ICU time	4.95 (0.02–136.03)	4.895 (0.04–136.03)	5.12 (0.03–101.73)	4.995 (0.02–81.28)	4.695 (0.12–63.93)	0.259
ICU dead	1,114 (20.57%)	180 (13.29%)	249 (18.39%)	307 (22.67%)	378 (27.92%)	<0.001

AKI, acute kidney injury; CKD, chronic kidney disease; HLD, hyperlipidemia; CB, chronic bronchitis; HF, heart failure; MI, myocardial infarction; IHD, ischemic heart disease; COPD, chronic obstructive pulmonary disease; BMI, body mass index; SOFA, sequential organ failure assessment; APSIII, acute physiology and chronic health III score; sirs, systemic inflammatory response syndrome score; Charlson, Charlson’s comorbidity index score; SAPSII, simplified acute physiology score II; OASIS, oxford acute severity of illness score; APACHII, acute physiology and chronic health evaluation II; HR, heart rate; NBPS, non-invasive blood pressure systolic; RR, respiratory rate; NBPD, non-invasive diastolic blood pressure; SPO2, oxygen saturation; HCT, hematocrit; HB, hemoglobin; PLT, platelet; RDW, red blood cell distribution width; RBC, red blood cell count; WBC, white blood cell; ALB, albumin; AG, anion gap; GLU, glucose; K, blood potassium; NA, blood sodium; MG, blood magnesium; TCO2, total amount of carbon dioxide; PCO2, partial pressure of carbon dioxide; LAC, lactate; PH, acidity and alkalinity; PO2, oxygen partial pressure; INR, international normalized ratio; PT, prothrombin time; APTT, activated partial thromboplastin time; ALT, alanine aminotransferase; AST, aspartate aminotransferase; TB, total bilirubin; CRE, creatinine; BUN, urea nitrogen; CRRT, continuous renal replacement therapy; ventilation, mechanical ventilation; GC, corticosteroids; ABX, antibiotics; VP, vasoactive drugs; SA, analgesic and sedative drugs.

### Multivariable cox regression analysis of the association between EASIX and short-term mortality in patients with pulmonary sepsis

**Figure 2:**
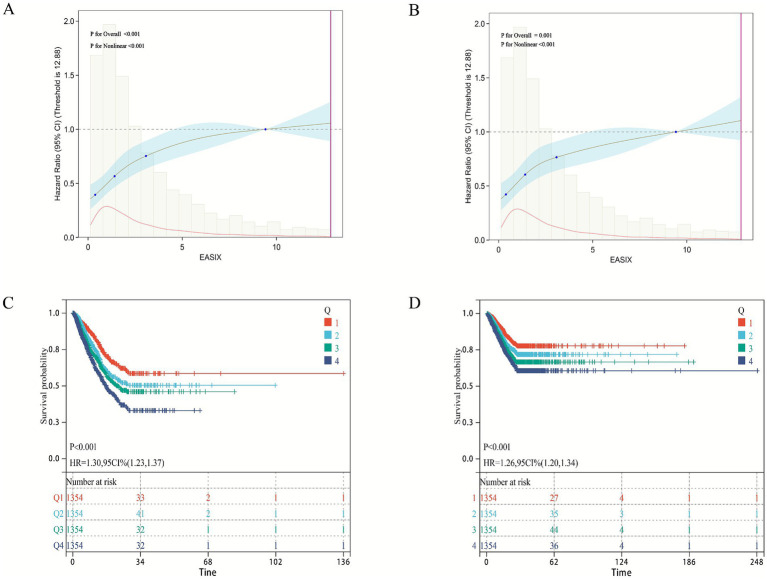
Association between EASIX and 28-day survival in the internal cohort. **(A,B)** Restricted cubic spline plots showing the non-linear relationship between the EASIX score and the adjusted hazard ratio (HR) for **(A)** 28-day ICU mortality and **(B)** 28-day in-hospital mortality. Solid lines represent the adjusted HR, and shaded areas indicate 95% confidence intervals. **(C,D)** Kaplan–Meier survival curves for **(C)** ICU survival and **(D)** in-hospital survival, stratified by EASIX quartiles. The hazard ratio (HR) and 95% confidence interval for the highest quartile (Q4) versus the lowest (Q1) are derived from the fully adjusted Cox model (Model 3).

As shown in Table 2, both continuous and categorical EASIX were significantly associated with 28-day ICU and in-hospital mortality in patients with pulmonary sepsis across all models (Models 1–3). The proportional hazards assumption was verified using Schoenfeld residuals. The global test for the fully adjusted ICU mortality model (Model 3) yielded *p* = 0.098, indicating no strong evidence against the proportionality assumption for the model as a whole. Secondly, EASIX as the continuity and categorical variables in the primary and secondary outcomes (28 day ICU/hospital death), and found that regardless of the type of variable EASIX used, its *p*-value was>0.05 (Supplementary Figure S5), confirming that the proportional hazards assumption held for our key predictor. In the fully adjusted model (Model 3), each unit increase in continuous EASIX was associated with a 7% higher risk of both ICU (HR 1.07, 95% CI 1.05–1.11) and in-hospital mortality (HR 1.07, 95% CI 1.04–1.09). When analyzed by quartiles, patients in the highest EASIX group (Q4) had nearly twice the risk of ICU death (HR 1.99, 95% CI 1.60–2.46) and an 81% higher risk of in-hospital death (HR 1.81, 95% CI 1.46–2.26) compared to the lowest quartile (Q1). Trend analysis further confirmed a significant dose–response relationship between increasing EASIX quartiles and both 28-day ICU and in-hospital mortality (*p* < 0.05). Restricted cubic spline analysis revealed a significant non-linear relationship between EASIX and mortality (*p* for non-linearity < 0.05; Figures 2A,B), and Kaplan–Meier survival curves visually corroborated the graded increase in mortality risk with higher EASIX scores (Figures 2C,D).

**Table 2:** The relationship between EASIX score and 28 day ICU/hospital mortality rate.

	Model 1			Model 2			Model 3		
Characteristic	HR^1^	95% CI^**1**^	p-value	HR^1^	95% CI^**1**^	*p*-value	HR^1^	95% CI^**1**^	*p*-value
28-day ICU mortality									
Continuous EASIX	1.09	1.07, 1.11	<0.001	1.08	1.06, 1.10	<0.001	1.07	1.05, 1.11	<0.001
EASIX group									
Q1	Ref	Ref		Ref	Ref		Ref	Ref	
Q2	1.39	1.14, 1.68	0.001	1.35	1.11,1.64	0.003	1.27	1.04, 1.55	0.019
Q3	1.72	1.43, 2.08	<0.001	1.70	1.40, 2.05	<0.001	1.61	1.32, 1.96	<0.001
Q4	2.23	1.89, 2.66	<0.001	2.13	1.76, 2.57	<0.001	1.99	1.60, 2.46	<0.001
28-day in-hospital mortality									
Continuous EASIX	1.08	1.06, 1.11	<0.001	1.09	1.06 1.11	<0.001	1.07	1.04, 1.09	<0.001
EASIX group									
Q1	Ref	Ref		Ref	Ref		Ref	Ref	
Q2	1.44	1.20, 1.73	<0.001	1.31	1.07 1.60	0.009	1.26	1.03, 1.55	0.025
Q3	1.81	1.52, 2.16	<0.001	1.68	1.34, 2.04	<0.001	1.55	1.26, 1.90	<0.001
Q4	2.22	1.81, 2.67	<0.001	2.03	1.67, 2.46	<0.001	1.81	1.46, 2.26	<0.001

HR, hazard ratio; CI, confidence interval; EASIX, endothelial activation and stress index; EASIX group, grouping variables endothelial activation and stress. Model 1, unadjusted; Model 2, adjusted for demographic characteristics (age, gender, race, and weight) and baseline comorbidities; Model 3, further adjusted for variables that showed significant differences between survivors and non survivors (laboratory tests, vital sign indicators, disease severity score at admission, and treatment interventions).

### Subgroup analysis

**Figure 3:**
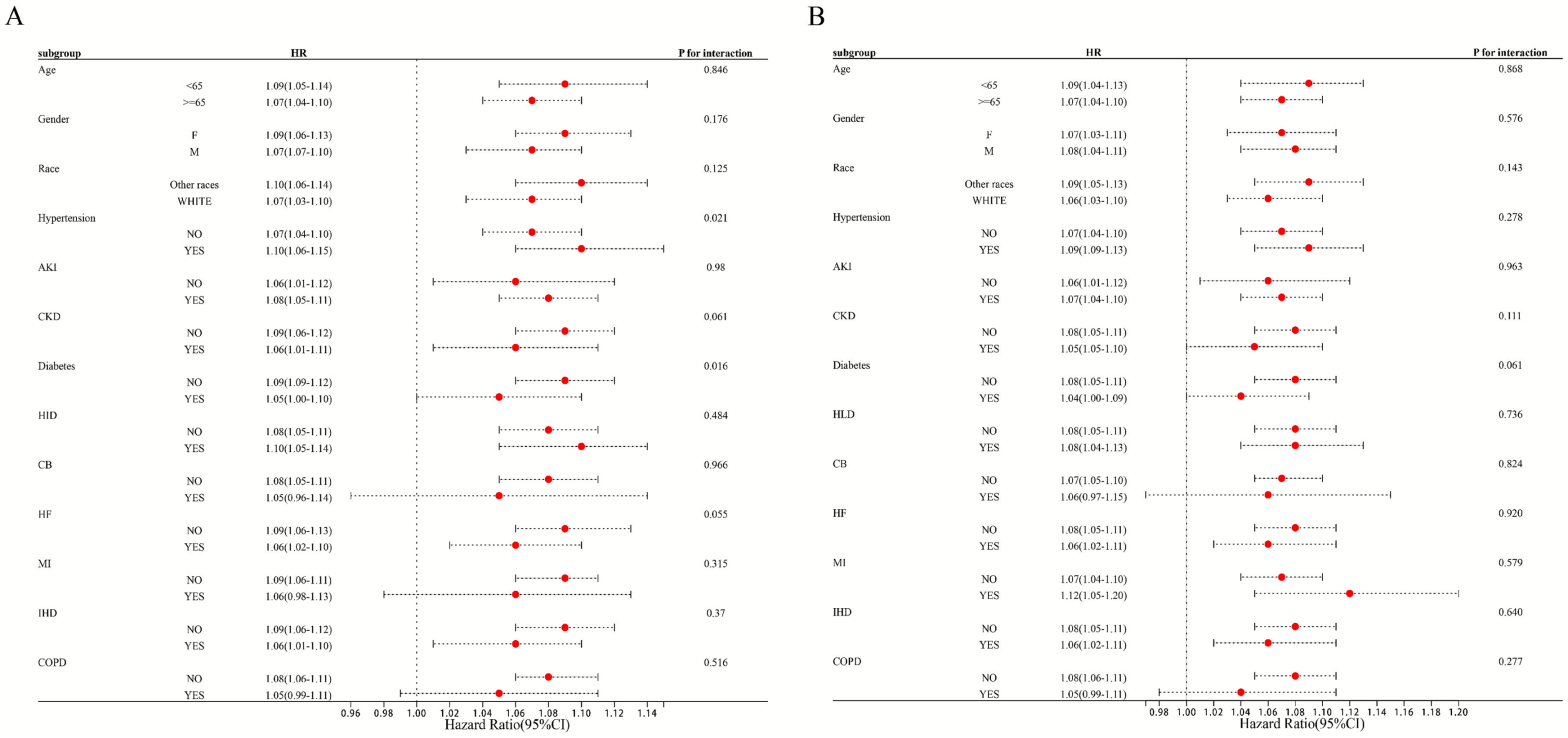
Subgroup analysis of EASIX and 28-day ICU/hospitalization mortality rate. Forest plot presenting fully adjusted hazard ratios (with 95% confidence intervals) per 1-unit increase in EASIX for 28-day ICU mortality (circles) and in-hospital mortality (squares) across patient subgroups. *P* for interaction values are from tests of the EASIX-by-subgroup interaction term in the Cox model.

Subgroup analyses were conducted to evaluate the consistency of EASIX prognostic performance across clinically relevant strata. Overall, elevated EASIX remained a significant predictor of both 28-day ICU and in-hospital mortality in nearly all subgroups examined, including those defined by age, sex, race, and most major comorbidities (Supplementary Tables S6, S7). These results confirm the generalizability of EASIX as a risk stratification tool beyond baseline patient characteristics. Interaction analysis revealed important nuances: hypertension and diabetes mellitus emerged as statistically significant effect modifiers for ICU mortality (*P* for interaction = 0.021 and 0.016, respectively). In patients with hypertension, the mortality risk associated with elevated EASIX was more pronounced (HR 1.10) than in non-hypertensive patients (HR 1.07). Conversely, the association was slightly attenuated in patients with diabetes. These findings suggest that underlying metabolic and vascular conditions may modulate the risk captured by EASIX, highlighting populations where its interpretation might warrant particular attention. For other comorbidities (e.g., CKD, HF, COPD), no significant interaction was detected (Figure 3A). Similarly, in the analysis of in-hospital mortality, no subgroup showed significant effect modification, further supporting the robustness of the main association (Figure 3B).

### Boruta feature variable selection

Supplementary Figure 1 displays the results of the feature selection based on the Boruta algorithm. In the Boruta algorithm, features labeled as “Confirmed” are deemed more important than the shadow features and are considered useful predictors. Those labeled as “Rejected” are not more important than the shadow features and are typically considered irrelevant. Features marked as “Tentative” have unclear importance and may require further confirmation. Therefore, the variables in the orange area (“Confirmed”) in the figure were identified as important features, with a total of 27 variables related to 28-day ICU mortality being selected as feature variables. Among these, EASIX was identified as an important feature associated with 28-day ICU mortality.

### External cohort verification

**Figure 4:**
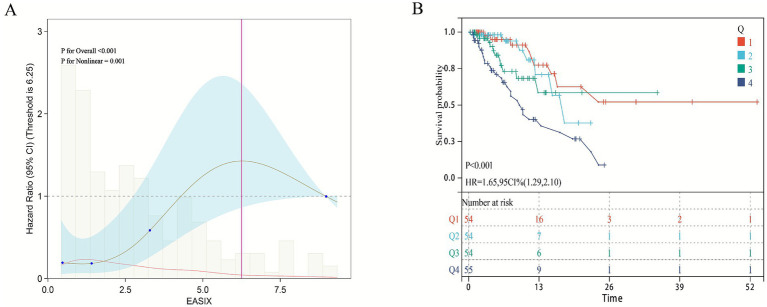
Association between EASIX and 28-day survival in the external validation cohort. **(A)** Restricted cubic spline plot showing the dose–response relationship between continuous Endothelial Activation and Stress Index (EASIX) values and 28-day in-hospital mortality in the external validation cohort. **(B)** Kaplan–Meier survival curves illustrate the probability of survival from ICU death 28 days, stratified by EASIX score stratification.

To further validate the relationship between EASIX and short-term mortality in patients with pulmonary sepsis, clinical data from 217 patients admitted to the Department of Pulmonary and Critical Care Medicine at Bijie Hospital of Zhejiang Provincial People’s Hospital between June 2023 and June 2025 were collected as an external validation cohort to verify the model’s effectiveness (Supplementary Tables S3, S4). The 28-day ICU mortality rate in this cohort was 24.35%. Notably, the fully adjusted multivariable Cox proportional hazards model (Model 3), which accounted for all potential covariates, showed that both continuous and categorical EASIX scores were significantly associated with 28-day ICU mortality (Table 3). For continuous EASIX, the hazard ratio was 1.13 (95% CI: 1.01–1.26). For categorical EASIX, although the comparison between Q2 and Q1 was not statistically significant, the highest EASIX group (Q4) showed a significantly increased risk of 28-day ICU mortality compared to the lowest group (Q1) (HR = 4.70, 95% CI: 1.54–14.38). Similarly, trend analysis confirmed a significant dose–response relationship between EASIX and 28-day ICU mortality (*p* < 0.001). Restricted Cubic Spline (RCS) analysis revealed a non-linear relationship between EASIX and short-term mortality in patients with pulmonary sepsis (non-linear *p* < 0.001) (Figure 4A). Kaplan–Meier survival curves confirmed a significant positive correlation between higher EASIX scores and increased 28-day ICU mortality (HR = 1.65, 95% CI: 1.29–2.10) (Figure 4B).

**Table 3:** The relationship between EASIX score and external verification cohort 28-day ICU mortality rate.

	Model1			Model2			Model3		
Characteristic	HR^1^	95% CI^1^	*p*-value	HR^1^	95% CI^1^	*p*-value	HR^1^	95% CI^1^	*p*-value
EASIX	1.16	1.08, 1.25	<0.001	1.13	1.05, 1.22	0.001	1.13	1.01, 1.26	0.031
EASIX group									
Q1	—	—		—	—		—	—	
Q2	1.36	0.51, 3.61	0.539	1.26	0.46, 3.46	0.650	1.24	0.39, 3.91	0.720
Q3	2.84	1.33, 6.07	0.007	3.63	1.62, 8.18	0.002	3.97	1.45, 10.84	0.007
Q4	4.61	2.10, 10.10	<0.001	4.40	1.88, 10.29	0.001	4.70	1.54, 14.38	0.047

HR, hazard ratio; CI, confidence interval; EASIX, endothelial activation and stress index; EASIX group, grouping variables endothelial activation and stress. Model 1, unadjusted; Model 2, adjusted for demographic characteristics (age, gender, race, and weight) and baseline comorbidities; Model 3, further adjusted for variables that showed significant differences between survivors and non survivors (laboratory tests, vital sign indicators, disease severity score at admission, and treatment interventions).

### Construction and validation of predictive models

**Figure 5:**
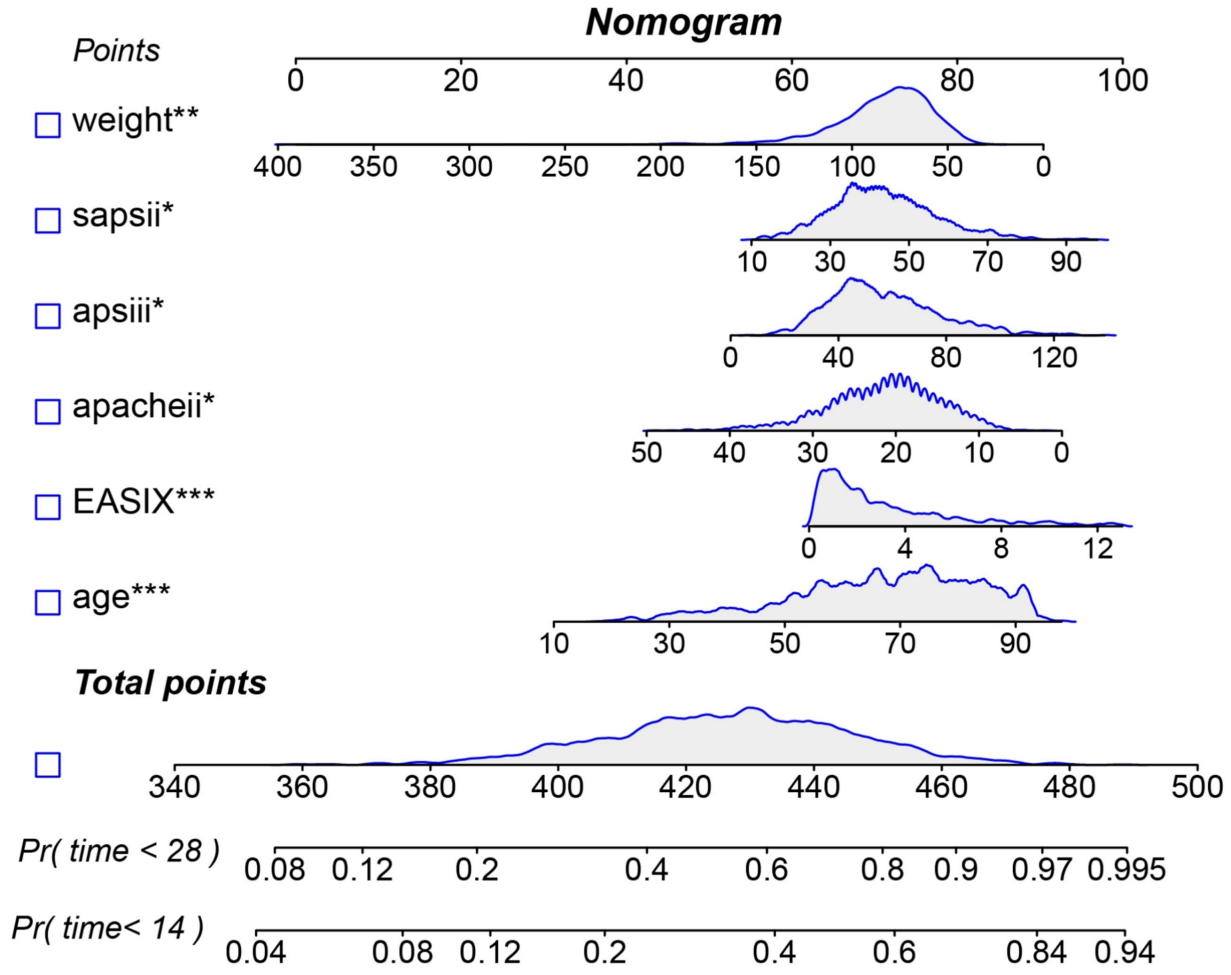
Nomogram for predicting the risk of 28-day ICU mortality. Note: This clinical tool integrates the EASIX score and other independent predictors identified from multivariate analysis. To use the nomogram: for each patient variable, locate the value on the corresponding axis and draw a line upward to the “Points” axis to determine the individual score. Sum the points for all variables, locate the total on the “Total Points” axis, and then draw a line straight down to the “Risk of 28-day ICU mortality” axis to obtain the individualized predicted probability.

**Figure 6:**
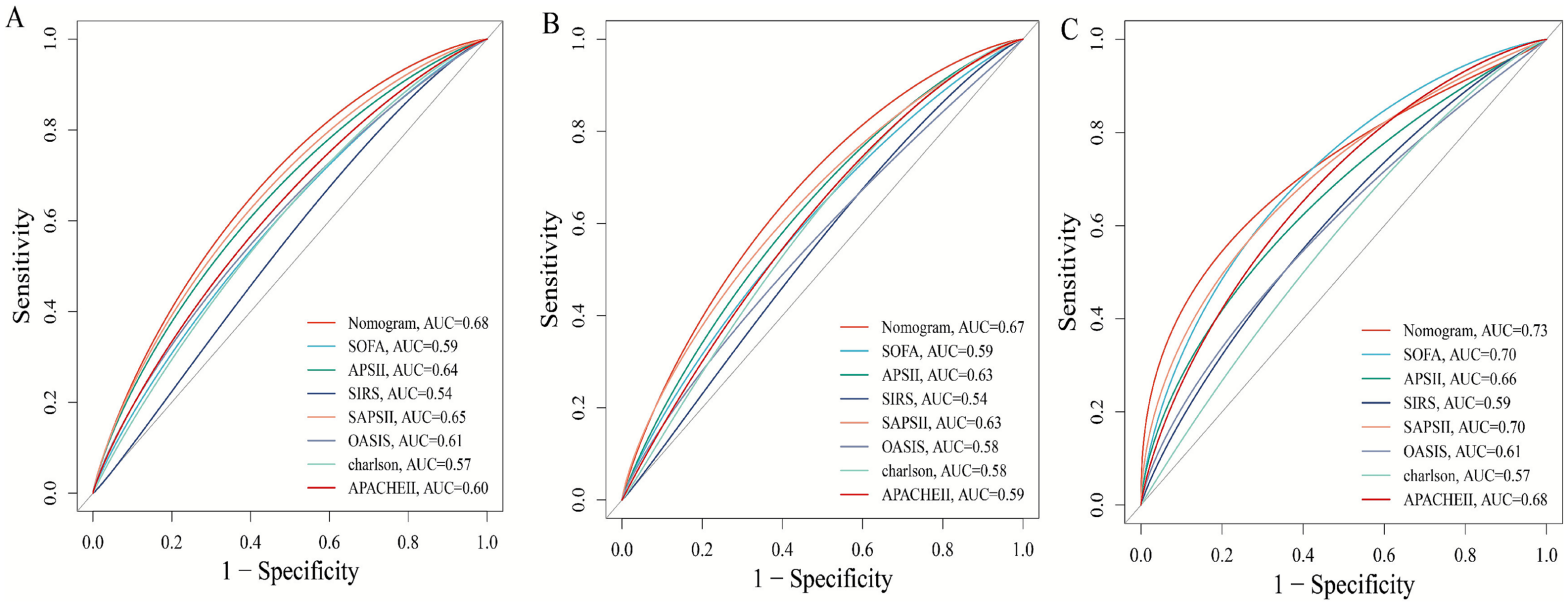
Receiver operating characteristic (ROC) curves comparing the predictive performance of the novel nomogram and conventional severity scores for 28-day mortality. **(A)** Test cohort 1; **(B)** train cohort 2; **(C)** external verification cohort.

In the internal cohort, all patients were randomly assigned to a training set and a test set in a 7:3 ratio. After excluding variables with collinearity (VIF > 5) and irrelevant variables removed by the Boruta algorithm, we evaluated the 27 core candidate variables in the training cohort using LASSO regression (Supplementary Figures S2A,B) and three machine learning methods: XGBoost, Gradient Boosting, and SVM. LASSO regression identified 19 key predictive variables, XGBoost identified 26 (Supplementary Figure S3A), Gradient Boosting identified 26 (Supplementary Figure S3B), and SVM identified 11 (Supplementary Figure S3C). We then used a Venn diagram to identify the common core prognostic variables determined by Boruta, LASSO, XGBoost, Gradient Boosting, and SVM (Supplementary Figure S4A), ultimately confirming six independent predictors: EASIX, age, APACHE II, APS III, SAPS II, and weight. Subsequently, we employed multivariable Cox regression to construct a risk prediction model for 28-day ICU mortality in patients with pulmonary sepsis (Supplementary Figure S4B). Compared to traditional severity scores (SOFA, APS III, SAPS II, OASIS, and APACHE II), the nomogram demonstrated higher sensitivity and specificity in predicting 28-day ICU mortality (Figure 5). The area under the receiver operating characteristic curve (AUROC) was 0.68 (test cohort; Figure 6A), 0.67 (training cohort; Figure 6B), and 0.73 (external validation cohort; Figure 6C).

## Discussion

This study, utilizing the large-scale public MIMIC-IV database and an external independent clinical cohort, is the first to comprehensively investigate and validate the prognostic value of the Endothelial Activation and Stress Index (EASIX) for short-term outcomes in patients with pulmonary sepsis. Our main findings can be summarized as follows: First, in patients with pulmonary sepsis, an elevated EASIX score was strongly associated with more critical clinical conditions, more severe organ dysfunction, and higher 28-day mortality. Second, multivariable Cox regression analysis demonstrated that EASIX is an independent risk factor for both 28-day ICU and in-hospital mortality, exhibiting a significant dose–response relationship. Third, subgroup analyses revealed that the predictive value of EASIX was consistent across various demographic groups; however, the strength of the association may be modified by specific comorbidities, such as hypertension and diabetes. Fourth, using advanced machine learning algorithms for feature selection, EASIX was identified as a core predictive variable. The EASIX-integrated nomogram prediction model constructed thereafter demonstrated superior discriminative ability compared to traditional severity scores and was robustly validated in the external cohort. Collectively, these results indicate that EASIX is a powerful, straightforward, and objective biomarker for assessing endothelial dysfunction and critical illness severity in pulmonary sepsis patients, holding significant potential for clinical translation. This is consistent with Xu et al.’s finding that elevated EASIX can predict 28 day mortality in a mixed cohort of sepsis ICU patients (24). The consistency of direction and significance in these studies emphasizes the fundamental role of endothelial dysfunction in sepsis outcomes.

Second, we observed that higher EASIX quartiles were associated with significantly increased disease severity scores (e.g., SOFA, APACHE II), indicating a clear correlation between EASIX and overall critical illness severity. More importantly, elevated EASIX levels showed strong consistency with biomarker changes indicative of endothelial and microvascular dysfunction: Decreased platelet count and haemoglobin suggest endothelial-mediated consumptive coagulopathy and microangiopathic haemolysis; elevated liver enzymes (ALT, AST), bilirubin, lactate, urea, and creatinine reflect concomitant hepatic and renal impairment along with tissue hypoperfusion following endothelial injury, while abnormal coagulation parameters (INR, PT, APTT) further indicate coagulation system activation due to endothelial dysfunction. These findings are consistent with the core pathological mechanism of sepsis – endothelial cell dysfunction (25, 26). In sepsis, pathogens and their toxins trigger a “cytokine storm,” leading to endothelial cell activation, apoptosis, and barrier disruption, which subsequently causes microcirculatory dysfunction, tissue oedema, coagulation activation, and multiple organ failure (27, 28). The core components of the EASIX formula (LDH, CRE, PLT) serve as sensitive indicators reflecting these processes: LDH marks cellular damage and haemolysis; CRE represents renal function (the kidneys being particularly vulnerable to microcirculatory impairment in sepsis); and decreased PLT is a characteristic feature of sepsis-associated coagulopathy (SAC) and endothelial consumption (29, 30). Therefore, as a composite index, EASIX effectively integrates multiple downstream effects of endothelial injury, enabling a comprehensive and quantitative assessment of systemic endothelial dysfunction severity.

Moreover, a notable strength of this study lies in its use of multiple machine learning algorithms (Boruta, LASSO, XGBoost, Gradient Boosting, and SVM) for high-dimensional variable screening, enabling data-driven and objective identification of key predictors. Among numerous clinical indicators and scores, EASIX was consistently identified as a “Confirmed” important feature associated with 28-day ICU mortality by all algorithms. Ultimately, it was selected, along with age, weight, APACHE II, SAPS II, and APS III, for inclusion in the final model. The nomogram prediction model constructed from these variables demonstrated good and stable predictive performance (AUC 0.67–0.73) across the internal test set, training set, and external validation cohort, outperforming any single traditional scoring system (e.g., SOFA, APACHE II). This finding holds significant clinical implications. First, it demonstrates that combining a biomarker reflecting a specific pathophysiological pathway (endothelial dysfunction, EASIX) with general condition assessment tools (traditional scores) can yield a more accurate and mechanistically informed prediction model. Second, since EASIX is calculated using routine laboratory parameters (LDH, creatinine, platelets) without additional costs, it can be easily obtained and automated at various levels of hospitals, offering high cost-effectiveness and scalability. This lays the groundwork for developing real-time, dynamic risk early-warning systems. Clinicians can use the EASIX value at admission or ICU entry to rapidly identify patients with pulmonary sepsis who appear stable but are actually at high risk, enabling earlier initiation of more intensive monitoring and interventions, such as closer microcirculation assessment, more cautious fluid management, or experimental therapeutic strategies targeting endothelial protection.

However, it must be acknowledged that this study has several limitations. First, as a retrospective observational study, although we rigorously adjusted for known confounding factors, the possibility of residual confounding or influence from unknown factors cannot be entirely ruled out. Second, EASIX is a static measurement; this study did not explore the relationship between its dynamic trends and patient outcomes. Serial monitoring of EASIX might better reflect disease progression and treatment response. Simultaneously, the prognostic nomogram we constructed, while demonstrating superior discriminative ability, incorporates several established composite severity scores (APACHE II, APS III, SAPS II). Although this design was necessary for our study to rigorously control for overall illness severity and to demonstrate the incremental value of EASIX over current standards, it may limit the model’s convenience for immediate manual calculation at the bedside. Future research should aim to validate and potentially simplify this tool, possibly by developing a points-based risk score using the core individual components (including EASIX) identified in this analysis, to enhance its practicality in resource-variable settings. Fourth, while external validation confirmed the prognostic significance of EASIX, the observed hazard ratio in the external cohort was higher than in the internal cohort. This discrepancy likely reflects differences in sample size, case-mix severity, and local clinical practices, underscoring the well-known challenge of transporting prediction models across sites. Therefore, our current nomogram should be considered a proof-of-concept model that establishes the value of incorporating EASIX. Formal evaluation of model calibration, stability (e.g., via bootstrapping), and potential recalibration for specific settings are necessary next steps before clinical deployment. Therefore, future multi-center, prospective studies are needed not only to validate our findings but also to address the natural heterogeneity between cohorts. Such studies should focus on assessing model calibration across diverse settings, exploring the potential for dynamic EASIX monitoring, and developing refined or simplified risk scores that maintain robust performance while enhancing clinical utility.

Finally, this study focused on prognostic prediction. Whether EASIX can serve as a target to guide therapy, and whether interventions targeting patients with high EASIX can improve outcomes, require further investigation through prospective interventional studies. The nomogram developed in this study, which integrates EASIX with established severity scores, demonstrated moderate discriminative ability (AUC 0.67–0.73) that was consistently superior to conventional scores such as SOFA and APACHE II. While this represents a statistically significant and clinically relevant incremental improvement, we acknowledge that its absolute performance is not yet sufficient for definitive, individual-level prognostication. The primary value of this model lies in its role as a proof-of-concept, validating that incorporating a specific marker of endothelial dysfunction (EASIX) into risk assessment adds meaningful information beyond general severity measures. To bridge the gap toward a higher-performance clinical tool, future research should focus on several strategies: Validating the utility of serial EASIX measurements to reflect treatment response and dynamic risk; secondly, combining EASIX with other emerging biomarkers of inflammation, coagulation, or organ injury to create a multi-panel predictor; and third, deriving and validating a simplified bedside score from the core variables identified here to improve usability.

## Conclusion

This study suggests that elevated EASIX is significantly associated with short-term mortality in critically ill patients with pulmonary sepsis. The combination of EASIX with age, APACHE II, APS III, SAPS II, and weight can effectively improve the accuracy of prognostic assessment, promote early identification of high-risk patients, and enable timely intervention. In addition, the non-linear relationship between EASIX and mortality provides a basis for personalized risk stratification, indicating that EASIX is a cost-effective biomarker with potential clinical value for optimizing risk assessment and treatment decisions in patients with severe pulmonary sepsis.

## Data Availability

The original contributions presented in the study are included in the article/Supplementary material, further inquiries can be directed to the corresponding author/s.

## References

[1] Vaughn VM Dickson RP Horowitz JK Flanders SA. Community-acquired pneumonia: a review. JAMA. (2024) 332:1282–95. doi: 10.1001/jama.2024.14796, 39283629

[2] Nair GB Niederman MS. Updates on community acquired pneumonia management in the ICU. Pharmacol Ther. (2021) 217:107663. doi: 10.1016/j.pharmthera.2020.107663, 32805298 PMC7428725

[3] Gu X Zhou F Wang Y Fan G Cao B. Respiratory viral sepsis: epidemiology, pathophysiology, diagnosis and treatment. Eur Respir Rev. (2020) 29:200038. doi: 10.1183/16000617.0038-2020 32699026 PMC9489194

[4] Gadsby NJ Musher DM. The microbial etiology of community-acquired pneumonia in adults: from classical bacteriology to host transcriptional signatures. Clin Microbiol Rev. (2022) 35:e0001522. doi: 10.1128/cmr.00015-22, 36165783 PMC9769922

[5] Aliberti S Dela Cruz CS Amati F Sotgiu G Restrepo MI. Community-acquired pneumonia. Lancet. (2021) 398:906–19. doi: 10.1016/S0140-6736(21)00630-9 34481570

[6] Vallecoccia MS Dominedò C Cutuli SL Martin-Loeches I Torres A De Pascale G. Is ventilated hospital-acquired pneumonia a worse entity than ventilator-associated pneumonia? Eur Respir Rev. (2020) 29:200023. doi: 10.1183/16000617.0023-2020 32759376 PMC9488552

[7] Liu YN Zhang YF Xu Q Qiu Y Lu QB Wang T . Infection and co-infection patterns of community-acquired pneumonia in patients of different ages in China from 2009 to 2020: a national surveillance study. Lancet Microbe. (2023) 4:e330–9. doi: 10.1016/S2666-5247(23)00031-9, 37001538 PMC12514336

[8] Luft T Benner A Jodele S Dandoy CE Storb R Gooley T . EASIX in patients with acute graft-versus-host disease: a retrospective cohort analysis. Lancet Haematol. (2017) 4:e414–23. doi: 10.1016/S2352-3026(17)30108-4, 28733186

[9] Lu J Wei Z Jiang H Cheng L Chen Q Chen M . Lactate dehydrogenase is associated with 28-day mortality in patients with sepsis: a retrospective observational study. J Surg Res. (2018) 228:314–21. doi: 10.1016/j.jss.2018.03.035, 29907227

[10] Chopra J Joist JH Webster RO. Loss of 51chromium, lactate dehydrogenase, and 111indium as indicators of endothelial cell injury. Lab Investig. (1987) 57:578–84. 3682767

[11] Maisons V Duval A Mesnard L Frimat M Fakhouri F Grangé S . Assessment of epidemiology and outcomes of adult patients with kidney-limited thrombotic microangiopathies. Kidney Int. (2024) 105:1100–12. doi: 10.1016/j.kint.2024.02.014 38431217

[12] Leisring J Brodsky SV Parikh SV. Clinical evaluation and management of thrombotic microangiopathy. Arthritis Rheumatol. (2024) 76:153–65. doi: 10.1002/art.42681, 37610060

[13] Baaten CCFMJ Nagy M Bergmeier W Spronk HMH van der Meijden PEJ. Platelet biology and function: plaque erosion vs. rupture. Eur Heart J. (2024) 45:18–31. doi: 10.1093/eurheartj/ehad720 37940193 PMC10757869

[14] Qiao X Yin J Zheng Z Li L Feng X. Endothelial cell dynamics in sepsis-induced acute lung injury and acute respiratory distress syndrome: pathogenesis and therapeutic implications. Cell Commun Signal. (2024) 22:241. doi: 10.1186/s12964-024-01620-y, 38664775 PMC11046830

[15] Sun R Huang J Sun B. Mobilization of endothelial progenitor cells in sepsis. Inflamm Res. (2020) 69:1–9. doi: 10.1007/s00011-019-01299-9, 31758219

[16] Evans CE Zhao YY. Impact of thrombosis on pulmonary endothelial injury and repair following sepsis. Am J Physiol Lung Cell Mol Physiol. (2017) 312:L441–51. doi: 10.1152/ajplung.00441.2016, 28130261 PMC5407094

[17] Xu H Sheng S Luo W Xu X Zhang Z. Acute respiratory distress syndrome heterogeneity and the septic ARDS subgroup. Front Immunol. (2023) 14:1277161. doi: 10.3389/fimmu.2023.1277161 38035100 PMC10682474

[18] Zhou T Long K Chen J Zhi L Zhou X Gao P. Global research progress of endothelial cells and ALI/ARDS: a bibliometric analysis. Front Physiol. (2024) 15:1326392. doi: 10.3389/fphys.2024.1326392 38774649 PMC11107300

[19] Merz A Germing U Kobbe G Kaivers J Jauch A Radujkovic A . EASIX for prediction of survival in lower-risk myelodysplastic syndromes. Blood Cancer J. (2019) 9:85. doi: 10.1038/s41408-019-0247-z 31712595 PMC6848148

[20] Finke D Hund H Frey N Luft T Lehmann LH. EASIX (endothelial activation and stress index) predicts mortality in patients with coronary artery disease. Clin Res Cardiol. (2025) 114:1008–18. doi: 10.1007/s00392-024-02534-y, 39256221 PMC12283470

[21] He Y Li Y Xiaojin Y Wu D Jiang W Xie X. Endothelial activation and stress index for prediction of mortality in asthma. Front Med. (2025) 12:1622944. doi: 10.3389/fmed.2025.1622944 PMC12283289 40703256

[22] Ulrich H Behrend P Wiedekopf J Drenkhahn C Kock-Schoppenhauer AK Ingenerf J. Hands on the medical informatics initiative Core data set - lessons learned from converting the MIMIC-IV. Stud Health Technol Inform. (2021) 283:119–26. doi: 10.3233/SHTI210549 34545827

[23] Yue S Li S Huang X Liu J Hou X Zhao Y . Machine learning for the prediction of acute kidney injury in patients with sepsis. J Transl Med. (2022) 20:215. doi: 10.1186/s12967-022-03364-0, 35562803 PMC9101823

[24] Xu HB Ye Y Xue F Wu J Suo Z Zhang H. Association between endothelial activation and stress index and 28-day mortality in septic ICU patients: a retrospective cohort study. Int J Med Sci. (2023) 20:1165–73. 37575274 10.7150/ijms.85870 PMC10416722

[25] Joffre J Hellman J Ince C Ait-Oufella H. Endothelial responses in Sepsis. Am J Respir Crit Care Med. (2020) 202:361–70. doi: 10.1164/rccm.201910-1911TR 32101446

[26] Zhang H Wang Y Qu M Li W Wu D Cata JP . Neutrophil, neutrophil extracellular traps and endothelial cell dysfunction in sepsis. Clin Transl Med. (2023) 13:e1170. doi: 10.7150/ijms.85870, 36629024 PMC9832433

[27] Tang F Zhao XL Xu LY Zhang JN Ao H Peng C. Endothelial dysfunction: pathophysiology and therapeutic targets for sepsis-induced multiple organ dysfunction syndrome. Biomed Pharmacother. (2024) 178:117180. doi: 10.1016/j.biopha.2024.117180, 39068853

[28] Joffre J Hellman J. Oxidative stress and endothelial dysfunction in Sepsis and acute inflammation. Antioxid Redox Signal. (2021) 35:1291–307. doi: 10.1089/ars.2021.0027, 33637016

[29] Zhou Y Qi M Yang M. Current status and future perspectives of lactate dehydrogenase detection and medical implications: a review. Biosensors. (2022) 12:1145 36551112 10.3390/bios12121145 PMC9775244

[30] Vincent JL Francois B Zabolotskikh I Daga MK Lascarrou JB Kirov MY . Effect of a recombinant human soluble Thrombomodulin on mortality in patients with Sepsis-associated coagulopathy: the SCARLET randomized clinical trial. JAMA. (2019) 321:1993–2002. doi: 10.1001/jama.2019.5358 31104069 PMC6547077

