# Supplementary material for: The endothelial activation and stress index as a predictor of 28-day mortality in pulmonary sepsis: a retrospective two-cohort analysis
Source: Front Med (Lausanne). 2026 Jan 27;13:1714682. doi: 10.3389/fmed.2026.1714682 (PMC12886393; doi:10.3389/fmed.2026.1714682)
Supplement: Supplementary file 1 [file Data_Sheet_1.pdf]

```r
#    Load all necessary packages
library(readxl)
library(dplyr)
library(tidyr)
library(ggplot2)
library(survival)
library(survminer)
library(rms)
library(MASS)
library(car)
library(corrplot)
library(caret)
library(glmnet)
library(randomForest)
library(Boruta)
library(xgboost)
library(e1071)
library(pROC)
library(rmda)
library(Hmisc)
library(mice)
library(foreach)
library(doParallel)
library(gridExtra)
library(patchwork)
library(knitr)
library(kableExtra)
library(ggpubr)
library(ggsci)
library(openxlsx)
library(timeROC)
library(broom)
library(VennDiagram)

data <- read_excel("MIMIC_data.xlsx", sheet = 1)
cat("Data structure:\n")
```

```
str(data)
cat("\nData dimensions:", dim(data), "\n")
missing_summary <- sapply(data, function(x) sum(is.na(x)))
missing_percentage <- sapply(data, function(x) mean(is.na(x)) * 100)
missing_df <- data.frame(
Variable = names(missing_summary),
Missing_Count = missing_summary,
Missing_Percentage = missing_percentage
)
cat("\nVariables with >30% missing data:\n")
print(missing_df[missing_df$Missing_Percentage > 30, ])
variables_to_keep <- missing_df$Variable[missing_df$Missing_Percentage <= 30]
data <- data[, variables_to_keep]
vars_to_impute <- missing_df$Variable[missing_df$Missing_Percentage > 0 &
missing_df$Missing_Percentage <= 30]
vars_to_impute <- intersect(vars_to_impute, colnames(data))
if (length(vars_to_impute) > 0) {
cat("\nPerforming multiple imputation for", length(vars_to_impute), "variables...\n")
imp_data <- mice(data[, vars_to_impute], m = 5, maxit = 10, method = 'pmm')
completed_data <- complete(imp_data, 1)
data[, vars_to_impute] <- completed_data
cat("Multiple imputation completed.\n")
}
data$EASIX <- (data$first_lactate_dehydrogenase_ld * data$first_creatinine) /
data$first_platelet_count
data$EASIX_quartile <- cut(data$EASIX,
breaks = quantile(data$EASIX, probs = c(0, 0.25, 0.5, 0.75, 1), na.rm = TRUE),
labels = c("Q1", "Q2", "Q3", "Q4"),
include.lowest = TRUE)
categorical_vars <- c("gender", "race", "htn", "aki", "ckd", "dm", "hld", "cb", "hf",
                      "mi", "ihd", "copd", "crrt", "ventilation", "sa", "gc", "vp",
                      "abx",                    "death_within_hosp_28days",
"death_within_icu_28days")
```

```
for(var in categorical_vars) {
  if(var %in% colnames(data)) {
    data[[var]] <- as.factor(data[[var]])
  }
}
data$death_hosp <- as.numeric(data$death_within_hosp_28days)
data$death_icu <- as.numeric(data$death_within_icu_28days)
data$surv_time_hosp <- data$hosp_day
data$surv_time_icu <- data$icu_day
data$surv_time_hosp_censored <- pmin(data$hosp_day, 28)
data$status_hosp <- ifelse(data$surv_time_hosp_censored == 28 & data$death_hosp
== 0, 0, data$death_hosp)
data$surv_time_icu_censored <- pmin(data$icu_day, 28)
data$status_icu <- ifelse(data$surv_time_icu_censored == 28 & data$death_icu == 0,
0, data$death_icu)
# Generate baseline feature table
generate_table1 <- function(data, group_var = "EASIX_quartile") {
  results <- data.frame()

  # 1. EASIX
  easix_stats <- data %>%
    group_by(!!sym(group_var)) %>%
    summarise(
      Median = median(EASIX, na.rm = TRUE),
      Q1 = quantile(EASIX, 0.25, na.rm = TRUE),
      Q3 = quantile(EASIX, 0.75, na.rm = TRUE),
      Min = min(EASIX, na.rm = TRUE),
      Max = max(EASIX, na.rm = TRUE)
    ) %>%
    mutate(
```

```
    Value = sprintf("%.3f (%.3f-%.3f)", Median, Min, Max),
    Variable = "EASIX",
    Level = NA
  )

# 2. Age
age_stats <- data %>%
  group_by(!!sym(group_var)) %>%
  summarise(
    Median = median(age, na.rm = TRUE),
    Min = min(age, na.rm = TRUE),
    Max = max(age, na.rm = TRUE)
  ) %>%
  mutate(
    Value = sprintf("%.0f (%.0f-%.0f)", Median, Min, Max),
    Variable = "Age",
    Level = NA
  )

# 3. Gender
gender_stats <- data %>%
  group_by(!!sym(group_var), gender) %>%
  summarise(Count = n()) %>%
  group_by(!!sym(group_var)) %>%
  mutate(
    Percentage = Count / sum(Count) * 100,
    Value = sprintf("%d (%.1f%%)", Count, Percentage)
  ) %>%
  rename(Level = gender) %>%
  mutate(Variable = "Gender")

# 4. Race
race_stats <- data %>%
  group_by(!!sym(group_var), race) %>%
  summarise(Count = n()) %>%
```

```
    group_by(!!sym(group_var)) %>%
    mutate(
      Percentage = Count / sum(Count) * 100,
      Value = sprintf("%d (%.1f%%)", Count, Percentage)
    ) %>%
    rename(Level = race) %>%
    mutate(Variable = "Race")

  # 5. Weight
  weight_stats <- data %>%
    group_by(!!sym(group_var)) %>%
    summarise(
      Median = median(weight, na.rm = TRUE),
      Min = min(weight, na.rm = TRUE),
      Max = max(weight, na.rm = TRUE)
    ) %>%
    mutate(
      Value = sprintf("%.1f (%.1f-%.1f)", Median, Min, Max),
      Variable = "Weight",
      Level = NA
    )

  # 6. comorbidity
  comorbidity_vars <- c("htm", "aki", "ckd", "dm", "hld", "cb", "hf", "mi", "ihd
", "copd")
  comorbidity_stats <- data.frame()

  for (var in comorbidity_vars) {
    if (var %in% colnames(data)) {
      var_stats <- data %>%
        group_by(!!sym(group_var), !!sym(var)) %>%
        summarise(Count = n()) %>%
        group_by(!!sym(group_var)) %>%
        mutate(
          Percentage = Count / sum(Count) * 100,
```

```
        Value = sprintf("%d (%.1f%%)", Count, Percentage)
      ) %>%
      rename(Level = !!sym(var)) %>%
      mutate(Variable = var)

    comorbidity_stats <- bind_rows(comorbidity_stats, var_stats)
  }
}

# 7. Severity rating
severity_vars <- c("sofa", "apsiii", "sirs", "sapsii", "oasis", "charlson", "apache
ii")
severity_stats <- data.frame()

for (var in severity_vars) {
  if (var %in% colnames(data)) {
    var_stats <- data %>%
      group_by(!!sym(group_var)) %>%
      summarise(
        Median = median(.data[[var]], na.rm = TRUE),
        Min = min(.data[[var]], na.rm = TRUE),
        Max = max(.data[[var]], na.rm = TRUE)
      ) %>%
      mutate(
        Value = sprintf("%.0f (%.0f-%.0f)", Median, Min, Max),
        Variable = toupper(var),
        Level = NA
      )

    severity_stats <- bind_rows(severity_stats, var_stats)
  }
}

# 8. vital signs
```

```
vital_vars <- c("first_hr", "first_nbps", "first_nbpd", "first_rr", "first_spo2", "fi
rst_temperaturef")
vital_stats <- data.frame()

for (var in vital_vars) {
  if (var %in% colnames(data)) {
    var_stats <- data %>%
      group_by(!!sym(group_var)) %>%
      summarise(
        Median = median(.data[[var]], na.rm = TRUE),
        Min = min(.data[[var]], na.rm = TRUE),
        Max = max(.data[[var]], na.rm = TRUE)
      ) %>%
      mutate(
        Value = sprintf("%.1f (%.1f-%.1f)", Median, Min, Max),
        Variable = var,
        Level = NA
      )

    vital_stats <- bind_rows(vital_stats, var_stats)
  }
}

# 9. Laboratory tests
lab_vars <- c("first_hematocrit", "first_hemoglobin", "first_platelet_count", "firs
t_rdw",
              "first_red_blood_cells", "first_white_blood_cells", "first_albumi
n", "first_anion_gap",
              "first_calcium_total", "first_chloride", "first_glucose", "first_pot
assium",
              "first_sodium", "first_calculated_total_co2", "first_lactate", "firs
t_pco2",
              "first_ph", "first_po2", "first_inrpt", "first_pt", "first_ptt",
              "first_alanine_aminotransferase_alt", "first_asparate_aminotransf
erase_ast",
```

```
                  "first_bilirubin_total", "first_creatinine", "first_urea_nitrogen",
                  "first_lactate_dehydrogenase_ld", "first_magnesium")

lab_stats <- data.frame()

for (var in lab_vars) {
  if (var %in% colnames(data)) {
    var_stats <- data %>%
      group_by(!!sym(group_var)) %>%
      summarise(
        Median = median(.data[[var]], na.rm = TRUE),
        Min = min(.data[[var]], na.rm = TRUE),
        Max = max(.data[[var]], na.rm = TRUE)
      ) %>%
      mutate(
        Value = sprintf("%.1f (%.1f-%.1f)", Median, Min, Max),
        Variable = var,
        Level = NA
      )

    lab_stats <- bind_rows(lab_stats, var_stats)
  }
}

#treatment measures
treatment_vars <- c("crrt", "ventilation", "sa", "gc", "vp", "abx")
treatment_stats <- data.frame()

for (var in treatment_vars) {
  if (var %in% colnames(data)) {
    var_stats <- data %>%
      group_by(!!sym(group_var), !!sym(var)) %>%
      summarise(Count = n()) %>%
      group_by(!!sym(group_var)) %>%
      mutate(
```

```
      Percentage = Count / sum(Count) * 100,
      Value = sprintf("%d (%.1f%%)", Count, Percentage)
    ) %>%
    rename(Level = !!sym(var)) %>%
    mutate(Variable = var)

  treatment_stats <- bind_rows(treatment_stats, var_stats)
 }
}

# outcome
outcome_stats <- data.frame()

# hosp_day
hosp_day_stats <- data %>%
 group_by(!!sym(group_var)) %>%
 summarise(
  Median = median(hosp_day, na.rm = TRUE),
  Min = min(hosp_day, na.rm = TRUE),
  Max = max(hosp_day, na.rm = TRUE)
 ) %>%
 mutate(
  Value = sprintf("%.2f (%.2f-%.2f)", Median, Min, Max),
  Variable = "hosp_day",
  Level = NA
 )

# hosp_dead
hosp_dead_stats <- data %>%
 group_by(!!sym(group_var), hosp_dead) %>%
 summarise(Count = n()) %>%
 group_by(!!sym(group_var)) %>%
 mutate(
  Percentage = Count / sum(Count) * 100,
  Value = sprintf("%d (%.1f%%)", Count, Percentage)
```

```
  ) %>%
  rename(Level = hosp_dead) %>%
  mutate(Variable = "hosp_dead")

# icu_day
icu_day_stats <- data %>%
  group_by(!!sym(group_var)) %>%
  summarise(
    Median = median(icu_day, na.rm = TRUE),
    Min = min(icu_day, na.rm = TRUE),
    Max = max(icu_day, na.rm = TRUE)
  ) %>%
  mutate(
    Value = sprintf("%.2f (%.2f-%.2f)", Median, Min, Max),
    Variable = "icu_day",
    Level = NA
  )

# icu_dead
icu_dead_stats <- data %>%
  group_by(!!sym(group_var), icu_dead) %>%
  summarise(Count = n()) %>%
  group_by(!!sym(group_var)) %>%
  mutate(
    Percentage = Count / sum(Count) * 100,
    Value = sprintf("%d (%.1f%%)", Count, Percentage)
  ) %>%
  rename(Level = icu_dead) %>%
  mutate(Variable = "icu_dead")

outcome_stats <- bind_rows(hosp_day_stats, hosp_dead_stats, icu_day_stats, ic
u_dead_stats)

all_stats <- bind_rows(
  easix_stats, age_stats, gender_stats, race_stats, weight_stats,
```

```
    comorbidity_stats, severity_stats, vital_stats, lab_stats,
    treatment_stats, outcome_stats
  )
  table_wide <- all_stats %>%
    select(Variable, Level, !!sym(group_var), Value) %>%
    pivot_wider(names_from = !!sym(group_var), values_from = Value) %>%
    mutate(Level = ifelse(is.na(Level), "", as.character(Level)))

  # Calculate P-value
  p_values <- data.frame()

  # P-value of continuous variable
  continuous_vars <- c("EASIX", "age", "weight", "sofa", "apsiii", "sirs", "sapsii
", "oasis",
                       "charlson", "apacheii", "first_hr", "first_nbps", "first_nb
pd", "first_rr",
                       "first_spo2", "first_temperaturef", "first_hematocrit", "fi
rst_hemoglobin",
                       "first_platelet_count", "first_rdw", "first_red_blood_cells
", "first_white_blood_cells",
                       "first_albumin", "first_anion_gap", "first_calcium_total",
 "first_chloride",
                       "first_glucose", "first_potassium", "first_sodium", "first_
calculated_total_co2",
                       "first_lactate", "first_pco2", "first_ph", "first_po2", "firs
t_inrpt", "first_pt",
                       "first_ptt", "first_alanine_aminotransferase_alt", "first_as
parate_aminotransferase_ast",
                       "first_bilirubin_total", "first_creatinine", "first_urea_nitr
ogen",
                       "first_lactate_dehydrogenase_ld", "first_magnesium", "h
osp_day", "icu_day")

  for (var in continuous_vars) {
    if (var %in% colnames(data)) {
```

```
        formula <- as.formula(paste(var, "~", group_var))
        kw_test <- kruskal.test(formula, data = data)
        p_values <- bind_rows(p_values, data.frame(Variable = var, P_value = k
w_test$p.value))
      }
    }

    # P-value of categorical variables
    for (var in categorical_vars) {
      if (var %in% colnames(data)) {
        contingency_table <- table(data[[group_var]], data[[var]])
        expected_counts <- chisq.test(contingency_table)$expected
        min_expected <- min(expected_counts)

        if (min_expected >= 5) {
          chi_test <- chisq.test(contingency_table)
          p_value <- chi_test$p.value
        } else {
          fisher_test <- fisher.test(contingency_table)
          p_value <- fisher_test$p.value
        }

        p_values <- bind_rows(p_values, data.frame(Variable = var, P_value = p
_value))
      }
    }

    final_table <- table_wide %>%
      left_join(p_values, by = "Variable") %>%
      mutate(
        P_value = ifelse(is.na(P_value), "",
                         ifelse(P_value < 0.001, "<0.001",
                                sprintf("%.3f", P_value))),
        Variable = ifelse(Level == "", Variable, paste0("   ", Level))
      ) %>%
```

```
    select(Variable, Q1, Q2, Q3, Q4, P_value)

  return(final_table)}
table1 <- generate_table1(data, "EASIX_quartile")
# Add variable labels
variable_labels <- c(
  "EASIX" = "EASIX",
  "age" = "Age",
  "gender" = "Gender",
  "race" = "Race",
  "weight" = "Weight",
  "htm" = "Hypertension",
  "aki" = "AKI",
  "ckd" = "CKD",
  "dm" = "Diabetes",
  "hld" = "HLD",
  "cb" = "CB",
  "hf" = "HF",
  "mi" = "MI",
  "ihd" = "IHD",
  "copd" = "COPD",
  "sofa" = "SOFA",
  "apsiii" = "APSIII",
  "sirs" = "SIRS",
  "sapsii" = "SAPSII",
  "oasis" = "OASIS",
  "charlson" = "charlson",
  "apacheii" = "APACHEII",
  "first_hr" = "HR",
  "first_nbps" = "NBPS",
  "first_nbpd" = "NBPD",
  "first_rr" = "RR",
  "first_spo2" = "Spo2",
  "first_temperaturef" = "Temperaturef (°F)",
  "first_hematocrit" = "HCT",
```

```
"first_hemoglobin" = "Hb",
"first_platelet_count" = "PLT",
"first_rdw" = "RDW",
"first_red_blood_cells" = "RBC",
"first_white_blood_cells" = "WBC",
"first_albumin" = "ALB",
"first_anion_gap" = "AG",
"first_calcium_total" = "Ca",
"first_chloride" = "Cl",
"first_glucose" = "GLU",
"first_potassium" = "K",
"first_sodium" = "Na",
"first_calculated_total_co2" = "TCO2",
"first_lactate" = "Lac",
"first_pco2" = "pco2",
"first_ph" = "ph",
"first_po2" = "po2",
"first_inrpt" = "INR",
"first_pt" = "PT",
"first_ptt" = "APTT",
"first_alanine_aminotransferase_alt" = "ALT",
"first_asparate_aminotransferase_ast" = "AST",
"first_bilirubin_total" = "TB",
"first_creatinine" = "CRE",
"first_urea_nitrogen" = "BUN",
"first_lactate_dehydrogenase_ld" = "LDH",
"first_magnesium" = "Mg",
"crrt" = "CRRT",
"ventilation" = "Ventilation",
"sa" = "Sa",
"gc" = "GC",
"vp" = "VP",
"abx" = "ABX",
"hosp_day" = "Hosp time",
"hosp_dead" = "Hosp dead",
```

```
  "icu_day" = "ICU time",
  "icu_dead" = "ICU dead")
for (i in 1:nrow(table1)) {
  var_name <- table1$Variable[i]
  if (var_name %in% names(variable_labels)) {
    table1$Variable[i] <- variable_labels[var_name]
  } else if (gsub("^  ", "", var_name) %in% names(variable_labels)) {
    table1$Variable[i] <- paste0("  ", variable_labels[gsub("^  ", "", var_nam
e)])
  }}
write.csv(table1, "Table1_Baseline_Characteristics.csv", row.names = FALSE)
```

#Kaplan-Meier survival curves

```
# ICU 28-day mortality

km_fit_icu <- survfit(Surv(surv_time_icu_censored, status_icu) ~ EASIX_quartile,
data = data)


# Hospital 28-day mortality

km_fit_hosp <- survfit(Surv(surv_time_hosp_censored, status_hosp) ~
EASIX_quartile, data = data)


# Plot Kaplan-Meier curves

km_plot_icu <- ggsurvplot(km_fit_icu,

                          data = data,

                          pval = TRUE,

                          pval.method = TRUE,

                          conf.int = TRUE,

                          risk.table = TRUE,

                          surv.median.line = "hv",
```

```
                                    palette = "jco",
                                    legend.labs = levels(data$EASIX_quartile),
                                    title = "Kaplan-Meier Curve for 28-day ICU
Mortality",
                                    xlab = "Time (days)",
                                    ylab = "Survival Probability")

km_plot_hosp <- ggsurvplot(km_fit_hosp,
                                     data = data,
                                     pval = TRUE,
                                     pval.method = TRUE,
                                     conf.int = TRUE,
                                     risk.table = TRUE,
                                     surv.median.line = "hv",
                                     palette = "jco",
                                     legend.labs = levels(data$EASIX_quartile),
                                     title = "Kaplan-Meier Curve for 28-day Hospital
Mortality",
                                     xlab = "Time (days)",
                                     ylab = "Survival Probability")

# Save plots
ggsave("KM_ICU_Mortality.png", plot = km_plot_icu$plot, width = 10, height = 8,
dpi = 300)
ggsave("KM_Hospital_Mortality.png", plot = km_plot_hosp$plot, width = 10, height
= 8, dpi = 300)
dev.off()
```

Restricted cubic spline analysis

```
# Set up data for rms package

dd <- datadist(data)

options(datadist = "dd")


# RCS analysis using continuous EASIX

rcs_model_icu <- cph(Surv(surv_time_icu_censored, status_icu) ~ rcs(
                              EASIX + race + first_temperaturef + gc + mi +
ventilation + first_white_blood_cells + hld + first_nbps + first_ptt + first_rr +
first_glucose + first_po2 + weight + aki + crrt + dm + first_albumin +
first_calcium_total + first_magnesium + first_rdw + first_hr + vp + sapsii +
first_ph + first_calculated_total_co2 + first_bilirubin_total + first_urea_nitrogen +
first_anion_gap + first_lactate + age + oasis + sofa + apacheii + apsiii + charlson,

                             data = data, x = TRUE, y = TRUE)


rcs_model_hosp <- cph(Surv(surv_time_hosp_censored, status_hosp) ~ rcs(EASIX +
race + first_temperaturef +
first_white_blood_cells + first_ptt + ventilation + first_nbps + first_glucose +
first_po2 + first_rr + weight + aki + crrt + dm + first_magnesium + first_rdw +
vp + first_albumin + first_hr + sa + first_ph + first_bilirubin_total +
first_calculated_total_co2 + first_urea_nitrogen + charlson + first_calcium_total +
first_anion_gap + first_lactate + age + oasis + sofa + apacheii + apsiii + sapsii,
                              data = data, x = TRUE, y = TRUE)


# Plot RCS

pdf("RCS_Plots.pdf", width = 10, height = 8)


# ICU mortality RCS

par(mfrow = c(1, 2))

plot(Predict(rcs_model_icu, EASIX, fun = exp),
```

```
        main = "RCS Analysis of EASIX on ICU Mortality",
        xlab = "EASIX", ylab = "Hazard Ratio")
abline(h = 1, lty = 2, col = "red")

# Hospital mortality RCS
plot(Predict(rcs_model_hosp, EASIX, fun = exp),
        main = "RCS Analysis of EASIX on Hospital Mortality",
        xlab = "EASIX", ylab = "Hazard Ratio")
abline(h = 1, lty = 2, col = "red")

dev.off()
```

#Cox proportional hazards regression

```
time_icu = pmin(icu_day, 28),
event_icu = death_within_icu_28days,
time_hosp = pmin(hosp_day, 28),
event_hosp = death_within_hosp_28days,

# Model 1: Unadjusted
cox_model1_icu <- coxph(Surv(surv_time_icu_censored, status_icu) ~ EASIX_quartile, data = data)
cox_model1_hosp <- coxph(Surv(surv_time_hosp_censored, status_hosp) ~ EASIX_quartile, data = data)

# Model 2: Adjusted for demographics and comorbidities
formula_icu_model2 <- Surv(time_icu, event_icu) ~ EASIX + race + weight + aki + mi + hld + age
results_icu_model2 <- run_cox_model(formula_icu_model2, data, "icu")
```

```
formula_hosp_model2 <- Surv(time_hosp, event_hosp) ~ EASIX + race + weight +
aki + age
results_hosp_model2 <- run_cox_model(formula_hosp_model2, data, "hosp")
formula_hosp_model2_cat <- Surv(time_hosp, event_hosp) ~ EASIX_quartile + race
+ weight + aki + age
results_hosp_model2_cat <- run_cox_model(formula_hosp_model2_cat, data, "hosp")

# Model 3: Fully adjusted model

formula_icu_model3 <- Surv(time_icu, event_icu) ~ EASIX + race +
first_temperaturef + gc + mi +
ventilation + first_white_blood_cells + hld + first_nbps + first_ptt + first_rr +
first_glucose + first_po2 + weight + aki + crrt + dm + first_albumin +
first_calcium_total + first_magnesium + first_rdw + first_hr + vp + sapsii +
first_ph + first_calculated_total_co2 + first_bilirubin_total + first_urea_nitrogen +
first_anion_gap + first_lactate + age + oasis + sofa + apacheii + apsiii + charlson
formula_icu_model3_cat <- Surv(time_icu, event_icu) ~ EASIX_quartile + race +
first_temperaturef + gc + mi +
ventilation + first_white_blood_cells + hld + first_nbps + first_ptt + first_rr +
first_glucose + first_po2 + weight + aki + crrt + dm + first_albumin +
first_calcium_total + first_magnesium + first_rdw + first_hr + vp + sapsii +
first_ph + first_calculated_total_co2 + first_bilirubin_total + first_urea_nitrogen +
first_anion_gap + first_lactate + age + oasis + sofa + apacheii + apsiii + charlson

formula_hosp_model3 <- Surv(time_hosp, event_hosp) ~ EASIX + race +
first_temperaturef +
first_white_blood_cells + first_ptt + ventilation + first_nbps + first_glucose +
first_po2 + first_rr + weight + aki + crrt + dm + first_magnesium + first_rdw +
vp + first_albumin + first_hr + sa + first_ph + first_bilirubin_total +
first_calculated_total_co2 + first_urea_nitrogen + charlson + first_calcium_total +
first_anion_gap + first_lactate + age + oasis + sofa + apacheii + apsiii + sapsii
results_hosp_model3 <- run_cox_model(formula_hosp_model3, data, "hosp")
formula_hosp_model3_cat <- Surv(time_hosp, event_hosp) ~ EASIX_quartile + race
+ first_temperaturef +
first_white_blood_cells + first_ptt + ventilation + first_nbps + first_glucose +
```

```
first_po2 + first_rr + weight + aki + crrt + dm + first_magnesium + first_rdw +
vp + first_albumin + first_hr + sa + first_ph + first_bilirubin_total +
first_calculated_total_co2 + first_urea_nitrogen + charlson + first_calcium_total +
first_anion_gap + first_lactate + age + oasis + sofa + apacheii + apsiii + sapsii
results_hosp_model3_cat <- run_cox_model(formula_hosp_model3_cat, data, "hosp")

extract_easix_results <- function(results_df, easix_type = "continuous") {
if (easix_type == "continuous") {easix_row <- results_df[grep("^EASIX$",
results_df$Characteristic), ]
if (nrow(easix_row) == 0) {
return(data.frame(
Characteristic = "Continuous EASIX",
HR = NA, CI_lower = NA, CI_upper = NA, p_value = NA
))
}
return(easix_row)
} else {easix_rows <- results_df[grep("EASIX_quartile", results_df$Characteristic), ]
if (nrow(easix_rows) == 0) {
return(data.frame(
Characteristic = c("Q2 vs Q1", "Q3 vs Q1", "Q4 vs Q1"),
HR = NA, CI_lower = NA, CI_upper = NA, p_value = NA
))
}
easix_rows$Characteristic <- c("Q2 vs Q1", "Q3 vs Q1", "Q4 vs Q1")
return(easix_rows)
}
}
format_ci <- function(lower, upper, hr) {
if (is.na(hr) || is.na(lower) || is.na(upper)) {
return("")
}
paste0(format(round(hr, 2), nsmall = 2),
" (", format(round(lower, 2), nsmall = 2),
"-", format(round(upper, 2), nsmall = 2), ")")
}
```

```
extract_easix_for_table <- function(continuous_results, categorical_results) {
cont_easix <- extract_easix_results(continuous_results, "continuous")
cat_easix <- extract_easix_results(categorical_results, "categorical")
return(list(continuous = cont_easix, categorical = cat_easix))
}
icu_model1 <- extract_easix_for_table(results_icu_model1, results_icu_model1_cat)
icu_model2 <- extract_easix_for_table(results_icu_model2, results_icu_model2_cat)
icu_model3 <- extract_easix_for_table(results_icu_model3, results_icu_model3_cat)
hosp_model1 <- extract_easix_for_table(results_hosp_model1,
results_hosp_model1_cat)
hosp_model2 <- extract_easix_for_table(results_hosp_model2,
results_hosp_model2_cat)
hosp_model3 <- extract_easix_for_table(results_hosp_model3,
results_hosp_model3_cat)
table2 <- data.frame(
Characteristic = c(
"Continuous EASIX",
"EASIX group",
" Q1 (Reference)",
" Q2 vs Q1",
" Q3 vs Q1",
" Q4 vs Q1",
"",
"Continuous EASIX",
"EASIX group",
" Q1 (Reference)",
" Q2 vs Q1",
" Q3 vs Q1",
" Q4 vs Q1"
),
Model1_HR = c(
# 28-day ICU mortality
format_ci(icu_model1$continuous$CI_lower, icu_model1$continuous$CI_upper,
icu_model1$continuous$HR),
"",
```

```
"Ref",
format_ci(icu_model1$categorical$CI_lower[1],
icu_model1$categorical$CI_upper[1], icu_model1$categorical$HR[1]),
format_ci(icu_model1$categorical$CI_lower[2],
icu_model1$categorical$CI_upper[2], icu_model1$categorical$HR[2]),
format_ci(icu_model1$categorical$CI_lower[3],
icu_model1$categorical$CI_upper[3], icu_model1$categorical$HR[3]),
"",
# 28-day in-hospital mortality
format_ci(hosp_model1$continuous$CI_lower, hosp_model1$continuous$CI_upper,
hosp_model1$continuous$HR),
"",
"Ref",
format_ci(hosp_model1$categorical$CI_lower[1],
hosp_model1$categorical$CI_upper[1], hosp_model1$categorical$HR[1]),
format_ci(hosp_model1$categorical$CI_lower[2],
hosp_model1$categorical$CI_upper[2], hosp_model1$categorical$HR[2]),
format_ci(hosp_model1$categorical$CI_lower[3],
hosp_model1$categorical$CI_upper[3], hosp_model1$categorical$HR[3])
),
Model1_p = c(
# 28-day ICU mortality
ifelse(!is.na(icu_model1$continuous$p_value),
format.pval(icu_model1$continuous$p_value, digits = 3, eps = 0.001), ""),
"",
"",
ifelse(!is.na(icu_model1$categorical$p_value[1]),
format.pval(icu_model1$categorical$p_value[1], digits = 3, eps = 0.001), ""),
ifelse(!is.na(icu_model1$categorical$p_value[2]),
format.pval(icu_model1$categorical$p_value[2], digits = 3, eps = 0.001), ""),
ifelse(!is.na(icu_model1$categorical$p_value[3]),
format.pval(icu_model1$categorical$p_value[3], digits = 3, eps = 0.001), ""),
"",
# 28-day in-hospital mortality
```

```
ifelse(!is.na(hosp_model1$continuous$p_value),
format.pval(hosp_model1$continuous$p_value, digits = 3, eps = 0.001), ""),
"",
"",
ifelse(!is.na(hosp_model1$categorical$p_value[1]),
format.pval(hosp_model1$categorical$p_value[1], digits = 3, eps = 0.001), ""),
ifelse(!is.na(hosp_model1$categorical$p_value[2]),
format.pval(hosp_model1$categorical$p_value[2], digits = 3, eps = 0.001), ""),
ifelse(!is.na(hosp_model1$categorical$p_value[3]),
format.pval(hosp_model1$categorical$p_value[3], digits = 3, eps = 0.001), "")
),
Model2_HR = c(
# 28-day ICU mortality
format_ci(icu_model2$continuous$CI_lower, icu_model2$continuous$CI_upper,
icu_model2$continuous$HR),
"",
"Ref",
format_ci(icu_model2$categorical$CI_lower[1],
icu_model2$categorical$CI_upper[1], icu_model2$categorical$HR[1]),
format_ci(icu_model2$categorical$CI_lower[2],
icu_model2$categorical$CI_upper[2], icu_model2$categorical$HR[2]),
format_ci(icu_model2$categorical$CI_lower[3],
icu_model2$categorical$CI_upper[3], icu_model2$categorical$HR[3]),
"",
# 28-day in-hospital mortality
format_ci(hosp_model2$continuous$CI_lower, hosp_model2$continuous$CI_upper,
hosp_model2$continuous$HR),
"",
"Ref",
format_ci(hosp_model2$categorical$CI_lower[1],
hosp_model2$categorical$CI_upper[1], hosp_model2$categorical$HR[1]),
format_ci(hosp_model2$categorical$CI_lower[2],
hosp_model2$categorical$CI_upper[2], hosp_model2$categorical$HR[2]),
format_ci(hosp_model2$categorical$CI_lower[3],
hosp_model2$categorical$CI_upper[3], hosp_model2$categorical$HR[3])
```

```
),
Model2_p = c(
# 28-day ICU mortality
ifelse(!is.na(icu_model2$continuous$p_value),
format.pval(icu_model2$continuous$p_value, digits = 3, eps = 0.001), ""),
"",
"",
ifelse(!is.na(icu_model2$categorical$p_value[1]),
format.pval(icu_model2$categorical$p_value[1], digits = 3, eps = 0.001), ""),
ifelse(!is.na(icu_model2$categorical$p_value[2]),
format.pval(icu_model2$categorical$p_value[2], digits = 3, eps = 0.001), ""),
ifelse(!is.na(icu_model2$categorical$p_value[3]),
format.pval(icu_model2$categorical$p_value[3], digits = 3, eps = 0.001), ""),
"",
# 28-day in-hospital mortality
ifelse(!is.na(hosp_model2$continuous$p_value),
format.pval(hosp_model2$continuous$p_value, digits = 3, eps = 0.001), ""),
"",
"",
ifelse(!is.na(hosp_model2$categorical$p_value[1]),
format.pval(hosp_model2$categorical$p_value[1], digits = 3, eps = 0.001), ""),
ifelse(!is.na(hosp_model2$categorical$p_value[2]),
format.pval(hosp_model2$categorical$p_value[2], digits = 3, eps = 0.001), ""),
ifelse(!is.na(hosp_model2$categorical$p_value[3]),
format.pval(hosp_model2$categorical$p_value[3], digits = 3, eps = 0.001), "")
),
Model3_HR = c(
# 28-day ICU mortality
format_ci(icu_model3$continuous$CI_lower, icu_model3$continuous$CI_upper,
icu_model3$continuous$HR),
"",
"Ref",
format_ci(icu_model3$categorical$CI_lower[1],
icu_model3$categorical$CI_upper[1], icu_model3$categorical$HR[1]),
```

```
format_ci(icu_model3$categorical$CI_lower[2],
icu_model3$categorical$CI_upper[2], icu_model3$categorical$HR[2]),
format_ci(icu_model3$categorical$CI_lower[3],
icu_model3$categorical$CI_upper[3], icu_model3$categorical$HR[3]),
"",
# 28-day in-hospital mortality
format_ci(hosp_model3$continuous$CI_lower, hosp_model3$continuous$CI_upper,
hosp_model3$continuous$HR),
"",
"Ref",
format_ci(hosp_model3$categorical$CI_lower[1],
hosp_model3$categorical$CI_upper[1], hosp_model3$categorical$HR[1]),
format_ci(hosp_model3$categorical$CI_lower[2],
hosp_model3$categorical$CI_upper[2], hosp_model3$categorical$HR[2]),
format_ci(hosp_model3$categorical$CI_lower[3],
hosp_model3$categorical$CI_upper[3], hosp_model3$categorical$HR[3])
),
Model3_p = c(
# 28-day ICU mortality
ifelse(!is.na(icu_model3$continuous$p_value),
format.pval(icu_model3$continuous$p_value, digits = 3, eps = 0.001), ""),
"",
"",
ifelse(!is.na(icu_model3$categorical$p_value[1]),
format.pval(icu_model3$categorical$p_value[1], digits = 3, eps = 0.001), ""),
ifelse(!is.na(icu_model3$categorical$p_value[2]),
format.pval(icu_model3$categorical$p_value[2], digits = 3, eps = 0.001), ""),
ifelse(!is.na(icu_model3$categorical$p_value[3]),
format.pval(icu_model3$categorical$p_value[3], digits = 3, eps = 0.001), ""),
"",
# 28-day in-hospital mortality
ifelse(!is.na(hosp_model3$continuous$p_value),
format.pval(hosp_model3$continuous$p_value, digits = 3, eps = 0.001), ""),
"",
"",
```

```
ifelse(!is.na(hosp_model3$categorical$p_value[1]),
format.pval(hosp_model3$categorical$p_value[1], digits = 3, eps = 0.001), ""),
ifelse(!is.na(hosp_model3$categorical$p_value[2]),
format.pval(hosp_model3$categorical$p_value[2], digits = 3, eps = 0.001), ""),
ifelse(!is.na(hosp_model3$categorical$p_value[3]),
format.pval(hosp_model3$categorical$p_value[3], digits = 3, eps = 0.001), "")
)
)
print(table2)
```

#VIF

```
icu_vars <- c(
"race", "first_temperaturef", "gc", "mi", "ventilation",
"first_white_blood_cells", "hld", "first_nbps", "first_ptt",
"first_rr", "first_glucose", "first_po2", "weight", "aki",
"crrt", "dm", "first_albumin", "first_calcium_total",
"first_magnesium", "first_rdw", "first_hr", "vp", "sapsii",
"first_ph", "first_calculated_total_co2", "first_bilirubin_total",
"first_urea_nitrogen", "first_anion_gap", "first_lactate", "age",
"oasis", "sofa", "apacheii", "apsiii", "charlson",
"first_asparate_aminotransferase_ast", "first_alanine_aminotransferase_alt",
"first_inrpt", "first_pt"
)
icu_formula <- as.formula(paste("first_temperaturef ~", paste(setdiff(icu_vars,
"first_temperaturef"), collapse = " + ")))
icu_model <- lm(icu_formula, data = icu_data)
icu_vif <- vif(icu_model)
icu_vif_df <- data.frame(
Variable_Names = names(icu_vif),
VIF = round(icu_vif, 2)
) %>%
arrange(desc(VIF)) %>%
mutate(Queue = "ICU-28Dead")
hosp_vars <- c(
"race", "first_temperaturef", "first_white_blood_cells",
```

```
"first_ptt", "ventilation", "first_nbps", "first_glucose",
"first_po2", "first_rr", "weight", "aki", "crrt", "dm",
"first_magnesium", "first_rdw", "vp", "first_albumin",
"first_hr", "sa", "first_ph", "first_bilirubin_total",
"first_calculated_total_co2", "first_urea_nitrogen",
"charlson", "first_calcium_total", "first_anion_gap",
"first_lactate", "age", "oasis", "sofa", "apacheii",
"apsiii", "sapsii", "first_asparate_aminotransferase_ast",
"first_alanine_aminotransferase_alt", "first_inrpt", "first_pt"
)
hosp_formula <- as.formula(paste("first_temperaturef ~", paste(setdiff(hosp_vars,
"first_temperaturef"), collapse = " + ")))
hosp_model <- lm(hosp_formula, data = hosp_data)
hosp_vif <- vif(hosp_model)
hosp_vif_df <- data.frame(
Variable_Names = names(hosp_vif),
VIF = round(hosp_vif, 2)
) %>%
arrange(desc(VIF)) %>%
mutate(Queue = "Hosp_28")
cat("Survival analysis completed.\n")
```

#subgroup analysis

```
data_subgroup <- data

data_subgroup$age_group <- factor(
  ifelse(data_subgroup$age < 65, "<65", ">=65"),
  levels = c("<65", ">=65"))

data_subgroup$race_group <- factor(
  ifelse(data_subgroup$race == "WHITE", "WHITE", "Other race"),
  levels = c("WHITE", "Other race"))

binary_vars <- c("htm", "aki", "ckd", "dm", "hld", "cb", "hf",
                 "mi", "ihd", "copd")
for (var in binary_vars) {
```

```
  if (var %in% colnames(data_subgroup)) {
    data_subgroup[[paste0(var, "_factor")]] <- factor(
      data_subgroup[[var]],
      levels = c(0, 1),
      labels = c("No", "Yes")
    )
  }}

perform_subgroup_analysis <- function(data, outcome = c("icu", "hosp")) {
  outcome <- match.arg(outcome)

  if (outcome == "icu") {
    time_var <- "time_icu"
    event_var <- "event_icu"
    outcome_name <- "28-day ICU mortality"
  } else {
    time_var <- "time_hosp"
    event_var <- "event_hosp"
    outcome_name <- "28-day hospital mortality"
  }

  model3_vars <- c(
    "race", "first_temperaturef", "first_white_blood_cells",
    "first_ptt", "ventilation", "first_nbps", "first_glucose",
    "first_po2", "first_rr", "weight", "aki", "crrt", "dm",
    "first_magnesium", "first_rdw", "vp", "first_albumin",
    "first_hr", "sa", "first_ph", "first_bilirubin_total",
    "first_calculated_total_co2", "first_urea_nitrogen",
    "charlson", "first_calcium_total", "first_anion_gap",
    "first_lactate", "age", "oasis", "sofa", "apacheii",
    "apsiii", "sapsii"
  )

  model3_vars <- model3_vars[model3_vars %in% colnames(data)]
```

```
# Define subgroup list
subgroup_list <- list(
  list(name = "Overall", var = NULL),
  list(name = "Age", var = "age_group"),
  list(name = "Gender", var = "gender"),
  list(name = "Race", var = "race_group"),
  list(name = "Hypertension", var = "htm_factor"),
  list(name = "AKI", var = "aki_factor"),
  list(name = "CKD", var = "ckd_factor"),
  list(name = "Diabetes", var = "dm_factor"),
  list(name = "Hyperlipidemia", var = "hld_factor"),
  list(name = "Chronic Bronchitis", var = "cb_factor"),
  list(name = "Heart Failure", var = "hf_factor"),
  list(name = "Myocardial Infarction", var = "mi_factor"),
  list(name = "Ischemic Heart Disease", var = "ihd_factor"),
  list(name = "COPD", var = "copd_factor")
)

results <- data.frame()

for (subgroup in subgroup_list) {
  subgroup_name <- subgroup$name
  subgroup_var <- subgroup$var

  if (is.null(subgroup_var)) {
    # Overall Analysis
    analysis_data <- data %>%
      select(all_of(c("EASIX", model3_vars, time_var, event_var))) %>%
      na.omit()

    formula_str <- paste("Surv(", time_var, ", ", event_var, ") ~ EASIX +",
                         paste(model3_vars, collapse = " + "))

    cox_model <- coxph(as.formula(formula_str), data = analysis_data)
    cox_summary <- summary(cox_model)
```

```
    if ("EASIX" %in% rownames(cox_summary$coefficients)) {
      easix_coef <- cox_summary$coefficients["EASIX", ]

      result_row <- data.frame(
        Subgroup = subgroup_name,
        Level = "Overall",
        N = nrow(analysis_data),
        Events = sum(analysis_data[[event_var]]),
        HR = exp(easix_coef["coef"]),
        Lower = exp(easix_coef["coef"] - 1.96 * easix_coef["se(coef)"]),
        Upper = exp(easix_coef["coef"] + 1.96 * easix_coef["se(coef)"]),
        P_value = easix_coef["Pr(>|z|)"],
        P_interaction = NA,
        stringsAsFactors = FALSE
      )

      results <- rbind(results, result_row)
    }

  } else {
    ####subgroup analysis
    subgroup_levels <- levels(data[[subgroup_var]])
    if (is.null(subgroup_levels)) {
      subgroup_levels <- unique(na.omit(data[[subgroup_var]]))
    }

    for (level in subgroup_levels) {
      analysis_data <- data %>%
        filter(.data[[subgroup_var]] == level) %>%
        select(all_of(c("EASIX", model3_vars, time_var, event_var))) %>%
        na.omit()

      if (nrow(analysis_data) < 50 || sum(analysis_data[[event_var]]) < 10) {
        next
```

```
      }

      formula_str <- paste("Surv(", time_var, ", ", event_var, ") ~ EASIX + 
",
                           paste(model3_vars, collapse = " + "))

      cox_model <- coxph(as.formula(formula_str), data = analysis_data)
      cox_summary <- summary(cox_model)

      if ("EASIX" %in% rownames(cox_summary$coefficients)) {
        easix_coef <- cox_summary$coefficients["EASIX", ]

        result_row <- data.frame(
          Subgroup = subgroup_name,
          Level = as.character(level),
          N = nrow(analysis_data),
          Events = sum(analysis_data[[event_var]]),
          HR = exp(easix_coef["coef"]),
          Lower = exp(easix_coef["coef"] - 1.96 * easix_coef["se(coef)"]),
          Upper = exp(easix_coef["coef"] + 1.96 * easix_coef["se(coef)"]),
          P_value = easix_coef["Pr(>|z|)"],
          P_interaction = NA,
          stringsAsFactors = FALSE
        )

        results <- rbind(results, result_row)
      }
    }

    # Interaction test
    if (length(subgroup_levels) > 1) {
      interaction_formula <- paste("Surv(", time_var, ", ", event_var, ") ~ E
ASIX *",
                                   subgroup_var, "+",
                                   paste(model3_vars, collapse = " + "))
```

```
          interaction_data <- data %>%
            select(all_of(c("EASIX", subgroup_var, model3_vars, time_var, event
_var))) %>%
            na.omit()

          interaction_model <- coxph(as.formula(interaction_formula),
                                      data = interaction_data)

          no_interaction_formula <- paste("Surv(", time_var, ", ", event_var, ")
~ EASIX +",
                                           subgroup_var, "+",
                                           paste(model3_vars, collapse = " +
 "))

          no_interaction_model <- coxph(as.formula(no_interaction_formula),
                                         data = interaction_data)

          lrt_test <- anova(no_interaction_model, interaction_model)
          p_interaction <- lrt_test[2, "P(>|Chi|)"]

          results$P_interaction[results$Subgroup == subgroup_name] <- p_interact
ion
        }
      }
    }

  results$Percent <- round(results$N / nrow(data) * 100, 1)
  results <- results %>%
    select(Subgroup, Level, N, Percent, everything())

  return(results)}
# Perform sub group analysis
icu_subgroup_results <- perform_subgroup_analysis(data_subgroup, outcome = "i
cu")
```

```
hosp_subgroup_results <- perform_subgroup_analysis(data_subgroup, outcome =
"hosp")

format_subgroup_table <- function(results, outcome_name) {
  formatted <- results %>%
    mutate(
      HR_CI = sprintf("%.2f (%.2f-%.2f)", HR, Lower, Upper),
      P_value = ifelse(P_value < 0.001, "<0.001",
                       ifelse(P_value < 0.01, sprintf("%.3f", P_value),
                              sprintf("%.3f", P_value))),
      P_interaction = ifelse(is.na(P_interaction), "",
                             ifelse(P_interaction < 0.001, "<0.001",
                                    sprintf("%.3f", P_interaction)))
    ) %>%
    select(Subgroup, Level, N, Percent, HR_CI, P_value, P_interaction)

  final_table <- data.frame(
    Variable = c(outcome_name, rep("", nrow(formatted))),
    Subgroup = c("Subgroup", formatted$Subgroup),
    Level = c("Level", formatted$Level),
    Count = c("Count", formatted$N),
    Percent = c("Percent", formatted$Percent),
    HR = c("HR (95% CI)", formatted$HR_CI),
    P_value = c("P-value", formatted$P_value),
    P_for_interaction = c("P for interaction", formatted$P_interaction)
  )

  return(final_table)}
table_s6 <- format_subgroup_table(icu_subgroup_results, "28-day ICU mortality")
table_s7 <- format_subgroup_table(hosp_subgroup_results, "28-day hospital morta
lity")

write.csv(table_s6, "Table_S6_ICU_Mortality_Subgroup_Analysis.csv", row.names
 = FALSE)
```

```
write.csv(table_s7, "Table_S7_Hospital_Mortality_Subgroup_Analysis.csv", row.na
mes = FALSE)

prepare_forestplot_data <- function(icu_results, hosp_results) {
  icu_filtered <- icu_results %>%
    filter(Subgroup != "Overall") %>%
    mutate(
      Outcome = "ICU Mortality",
      Subgroup_Level = ifelse(Level == "Overall", Subgroup, paste(Subgroup,
Level, sep = ": "))
    )

  hosp_filtered <- hosp_results %>%
    filter(Subgroup != "Overall") %>%
    mutate(
      Outcome = "Hospital Mortality",
      Subgroup_Level = ifelse(Level == "Overall", Subgroup, paste(Subgroup,
Level, sep = ": "))
    )

  combined_data <- bind_rows(icu_filtered, hosp_filtered) %>%
    arrange(Subgroup, Level) %>%
    mutate(row_id = row_number())

  return(combined_data)}

create_forest_plot <- function(data, title = "Subgroup Analysis of EASIX and 2
8-day Mortality") {
  p <- ggplot(data, aes(x = HR, y = reorder(Subgroup_Level, -row_id))) +
    geom_vline(xintercept = 1, linetype = "dashed", color = "gray50") +
    geom_point(aes(color = Outcome, shape = Outcome), size = 3,
               position = position_dodge(width = 0.5)) +
    geom_errorbarh(aes(xmin = Lower, xmax = Upper, color = Outcome),
                   height = 0.2, position = position_dodge(width = 0.5)) +
    scale_x_continuous(breaks = seq(0.5, 2.5, 0.5), limits = c(0.5, 2.5)) +
```

```
    scale_color_manual(values = c("ICU Mortality" = "#E69F00",
                                   "Hospital Mortality" = "#56B4E9")) +
    scale_shape_manual(values = c("ICU Mortality" = 19,
                                   "Hospital Mortality" = 17)) +
    labs(
      title = title,
      x = "Hazard Ratio (95% CI) per 1-unit increase in EASIX",
      y = "",
      color = "Outcome",
      shape = "Outcome"
    ) +
    theme_minimal() +
    theme(
      plot.title = element_text(hjust = 0.5, face = "bold", size = 14),
      axis.text.y = element_text(size = 10),
      axis.text.x = element_text(size = 10),
      axis.title.x = element_text(size = 11, face = "bold"),
      panel.grid.major = element_line(color = "gray90"),
      panel.grid.minor = element_blank(),
      legend.position = "bottom",
      legend.title = element_text(face = "bold")
    ) +
    facet_grid(Subgroup ~ ., scales = "free_y", space = "free_y") +
    theme(
      strip.background = element_rect(fill = "gray90", color = NA),
      strip.text.y = element_text(angle = 0, hjust = 0)
    )

  return(p)}

forestplot_data <- prepare_forestplot_data(icu_subgroup_results, hosp_subgroup_re
sults)
forest_plot <- create_forest_plot(forestplot_data)

ggsave("Figure3_Subgroup_Analysis_Forest_Plot.png", forest_plot,
```

```
        width = 14, height = 16, dpi = 300)
ggsave("Figure3_Subgroup_Analysis_Forest_Plot_HighRes.tiff", forest_plot,
        width = 14, height = 16, dpi = 600, compression = "lzw")

significant_interactions <- icu_subgroup_results %>%
  filter(!is.na(P_interaction) & P_interaction < 0.05) %>%
  distinct(Subgroup, P_interaction) %>%
  mutate(
    P_interaction = ifelse(P_interaction < 0.001, "<0.001",
                           sprintf("%.3f", P_interaction))
  )

interaction_summary <- data.frame(
  Subgroup = significant_interactions$Subgroup,
  `P for interaction` = significant_interactions$P_interaction,
  Interpretation = "Significant interaction between EASIX and this subgroup",
  stringsAsFactors = FALSE)

write.csv(interaction_summary, "Significant_Interactions_Summary.csv", row.name
s = FALSE)

generate_subgroup_report <- function(icu_results, hosp_results) {
  report <- list(
    ICU_Mortality = list(
      Overall = icu_results %>% filter(Subgroup == "Overall"),
      Subgroups = icu_results %>% filter(Subgroup != "Overall"),
      Significant_Interactions = icu_results %>%
        filter(!is.na(P_interaction) & P_interaction < 0.05) %>%
        distinct(Subgroup, P_interaction)
    ),
    Hospital_Mortality = list(
      Overall = hosp_results %>% filter(Subgroup == "Overall"),
      Subgroups = hosp_results %>% filter(Subgroup != "Overall"),
      Significant_Interactions = hosp_results %>%
        filter(!is.na(P_interaction) & P_interaction < 0.05) %>%
```

```
        distinct(Subgroup, P_interaction)
    ),
    Summary = list(
      Total_Patients = nrow(data_subgroup),
      ICU_Mortality_Rate = round(mean(data_subgroup$icu_dead, na.rm = TR
UE) * 100, 2),
      Hospital_Mortality_Rate = round(mean(data_subgroup$hosp_dead, na.rm =
 TRUE) * 100, 2),
      Number_of_Subgroups = length(unique(icu_results$Subgroup[icu_results$S
ubgroup != "Overall"])),
      Analysis_Date = Sys.Date()
    )
  )

  return(report)}

subgroup_report <- generate_subgroup_report(icu_subgroup_results, hosp_subgrou
p_results)

# Prepare data for machine learning

data <- read_excel("MIMIC_data.xlsx", sheet = 1)

#Create survival analysis variables

data$death_hosp <- as.numeric(data$death_within_hosp_28days)

data$death_icu <- as.numeric(data$death_within_icu_28days)


#Create a survival time variable (28 day truncation)

data$surv_time_hosp_censored <- pmin(data$hosp_day, 28)

data$status_hosp <- ifelse(data$surv_time_hosp_censored == 28 & data$death_hosp
== 0, 0, data$death_hosp)


data$surv_time_icu_censored <- pmin(data$icu_day, 28)
```

data$status_icu <- ifelse(data$surv_time_icu_censored == 28 & data$death_icu == 0, 0, data$death_icu)

Create simplified time variables for machine learning

data$time_icu <- pmin(data$icu_day, 28)

data$event_icu <- data$death_within_icu_28days

data$time_hosp <- pmin(data$hosp_day, 28)

data$event_hosp <- data$death_within_hosp_28days

#Convert categorical variables

categorical_vars <- c("gender", "race", "htm", "aki", "ckd", "dm", "hld", "cb", "hf",

"mi", "ihd", "copd", "crrt", "ventilation", "sa", "gc", "vp",

"abx", "death_within_hosp_28days", "death_within_icu_28days")

for (var in categorical_vars) {

if (var %in% colnames(data)) {

data[[var]] <- as.factor(data[[var]])

}

}

cat("Data preprocessing completed.\n")

#Divide the training set and testing set

set.seed(123)

```
train_indices <- createDataPartition(data$event_icu,
                                     p = 0.7,
                                     list = FALSE,
                                     times = 1)
```

```
train_data <- data[train_indices, ]
test_data <- data[-train_indices, ]

cat("Training set dimensions:", dim(train_data), "\n")
cat("Test set dimensions:", dim(test_data), "\n")

model3_vars <- c(
  "race", "first_temperaturef", "gc", "mi", "ventilation",
  "first_white_blood_cells", "hld", "first_nbps", "first_ptt",
  "first_rr", "first_glucose", "first_po2", "weight", "aki",
  "crrt", "dm", "first_albumin", "first_calcium_total",
  "first_magnesium", "first_rdw", "first_hr", "vp", "sapsii",
  "first_ph", "first_calculated_total_co2", "first_bilirubin_total",
  "first_urea_nitrogen", "first_anion_gap", "first_lactate", "age",
  "oasis", "sofa", "apacheii", "apsiii", "charlson",
  "first_asparate_aminotransferase_ast", "first_alanine_aminotransferase_alt",
  "EASIX")

train_data_boruta <- train_data %>%
  select(all_of(model3_vars), event_icu) %>%
  na.omit()
predictors_boruta <- train_data_boruta %>%
  select(-event_icu)

outcome_boruta <- train_data_boruta$event_icu
# Run Boruta algorithm
cat("Running Boruta algorithm...\n")
boruta_result <- Boruta(
  x = predictors_boruta,
  y = outcome_boruta,
  doTrace = 2,
  maxRuns = 100,
  getImp = getImpRfZ)
# Extract Boruta decision statistics
```

```
boruta_stats <- attStats(boruta_result)
boruta_stats$Variable <- rownames(boruta_stats)
# Remove shadow features
boruta_stats <- boruta_stats %>%
  filter(!Variable %in% c("shadowMax", "shadowMean", "shadowMin"))
# Create decision labels
boruta_stats$Decision <- factor(boruta_stats$decision,
                                levels = c("Confirmed", "Tentative", "Rejec
ted"),
                                labels = c("Confirmed", "Tentative", "Rejec
ted"))
# Sort by importance
boruta_stats <- boruta_stats %>%
  arrange(desc(meanImp))
# Create variable labels (with more user-friendly display names)
variable_labels <- list(
  "EASIX" = "EASIX",
  "age" = "Age",
  "race" = "Race",
  "first_temperaturef" = "Temperature",
  "gc" = "Glucocorticoids",
  "mi" = "Myocardial Infarction",
  "ventilation" = "Ventilation",
  "first_white_blood_cells" = "WBC",
  "hld" = "Hyperlipidemia",
  "first_nbps" = "Systolic BP",
  "first_ptt" = "PTT",
  "first_rr" = "Respiratory Rate",
  "first_glucose" = "Glucose",
  "first_po2" = "pO2",
  "weight" = "Weight",
  "aki" = "AKI",
  "crrt" = "CRRT",
  "dm" = "Diabetes",
  "first_albumin" = "Albumin",
```

```
  "first_calcium_total" = "Calcium",
  "first_magnesium" = "Magnesium",
  "first_rdw" = "RDW",
  "first_hr" = "Heart Rate",
  "vp" = "Vasopressors",
  "sapsii" = "SAPS II",
  "first_ph" = "pH",
  "first_calculated_total_co2" = "TCO2",
  "first_bilirubin_total" = "Bilirubin",
  "first_urea_nitrogen" = "BUN",
  "charlson" = "Charlson Index",
  "first_anion_gap" = "Anion Gap",
  "first_lactate" = "Lactate",
  "oasis" = "OASIS",
  "sofa" = "SOFA",
  "apacheii" = "APACHE II",
  "apsiii" = "APS III",
  "first_asparate_aminotransferase_ast" = "AST",
  "first_alanine_aminotransferase_alt" = "ALT")
# Apply variable labels
boruta_stats$Variable_Label <- boruta_stats$Variablefor (i in 1:nrow(boruta_stat
s)) {
  var_name <- boruta_stats$Variable[i]
  if (var_name %in% names(variable_labels)) {
    boruta_stats$Variable_Label[i] <- variable_labels[[var_name]]
  }}
# Create drawing data
plot_data <- boruta_stats %>%
  arrange(desc(meanImp)) %>%
  mutate(Variable_Label = factor(Variable_Label,
                                 levels = Variable_Label[order(meanImp)]))
# Create color mapping
decision_colors <- c(
  "Confirmed" = "#E69F00",
  "Tentative" = "#56B4E9",
```

```
  "Rejected" = "#999999")
figure <- ggplot(plot_data, aes(x = reorder(Variable_Label, meanImp), y = mean
Imp, fill = Decision)) +
  geom_bar(stat = "identity", width = 0.7) +
  coord_flip() +
  scale_fill_manual(
    values = decision_colors,
    name = "Boruta Decision",
    breaks = c("Confirmed", "Tentative", "Rejected"),
    labels = c("Confirmed (Important)", "Tentative", "Rejected (Irrelevant)")
  ) +
  labs(
    title = "Feature Selection Based on Boruta Algorithm",
    subtitle = "Importance of variables for predicting 28-day ICU mortality",
    x = "Variables",
    y = "Mean Importance (Z-score)",
    caption = paste("Total variables analyzed:", nrow(plot_data),
                    "\nConfirmed:", sum(plot_data$Decision == "Confirmed"),
                    "Tentative:", sum(plot_data$Decision == "Tentative"),
                    "Rejected:", sum(plot_data$Decision == "Rejected"))
  ) +
  theme_minimal(base_size = 12) +
  theme(
    plot.title = element_text(face = "bold", size = 16, hjust = 0.5),
    plot.subtitle = element_text(size = 12, hjust = 0.5, margin = margin(b = 1
0)),
    axis.title.x = element_text(face = "bold", margin = margin(t = 10)),
    axis.title.y = element_text(face = "bold", margin = margin(r = 10)),
    axis.text.y = element_text(size = 10, color = "black"),
    axis.text.x = element_text(size = 10, color = "black"),
    legend.position = "right",
    legend.title = element_text(face = "bold"),
    legend.text = element_text(size = 10),
    panel.grid.major = element_line(color = "gray90", size = 0.3),
    panel.grid.minor = element_blank(),
```

```
    plot.caption = element_text(size = 10, color = "gray50", hjust = 0, margin
 = margin(t = 10)),
    plot.margin = margin(20, 20, 20, 20)
  ) +
  geom_hline(yintercept = 0, color = "black", size = 0.5)
# Add tags for important variables
confirmed_vars <- plot_data %>%
  filter(Decision == "Confirmed") %>%
  arrange(desc(meanImp))
# Annotate on the diagram
EASIXif ("EASIX" %in% plot_data$Variable) {
  easix_row <- plot_data %>% filter(Variable == "EASIX")
  figure_s1 <- figure_s1 +
    geom_segment(
      data = easix_row,
      aes(x = Variable_Label, xend = Variable_Label,
          y = 0, yend = meanImp),
      color = "#D55E00",
      size = 1.2,
      arrow = arrow(length = unit(0.2, "cm"), type = "closed")
    ) +
    annotate("text",
             x = easix_row$Variable_Label,
             y = easix_row$meanImp + max(plot_data$meanImp) * 0.05,
             label = "EASIX (Confirmed)",
             color = "#D55E00",
             fontface = "bold",
             size = 4)}
ggsave(
  filename = "Figure.pdf",
  plot = figure_s1,
  width = 14,
  height = max(8, nrow(plot_data) * 0.3),
  device = "pdf")
```

#LASSO regression+three machine learning methods (XGBoost, Gradient Boosting, SVM) for feature filtering

```
model3_vars <- c(
  "race", "first_temperaturef", "gc", "mi", "ventilation",
  "first_white_blood_cells", "hld", "first_nbps", "first_ptt",
  "first_rr", "first_glucose", "first_po2", "weight", "aki",
  "crrt", "dm", "first_albumin", "first_calcium_total",
  "first_magnesium", "first_rdw", "first_hr", "vp", "sapsii",
  "first_ph", "first_calculated_total_co2", "first_bilirubin_total",
  "first_urea_nitrogen", "first_anion_gap", "first_lactate", "age",
  "oasis", "sofa", "apacheii", "apsiii", "charlson",
  "first_asparate_aminotransferase_ast", "first_alanine_aminotransferase_alt",
  "EASIX")
model3_vars <- model3_vars[model3_vars %in% colnames(train_data)]
feature_selection_data <- train_data %>%
  select(all_of(model3_vars), time_icu, event_icu) %>%
  na.omit()
#LASSO data preparation
X_lasso <- as.matrix(feature_selection_data[, model3_vars])
Y_lasso <- Surv(feature_selection_data$time_icu, feature_selection_data$event_icu)
set.seed(123)
lasso_cv <- cv.glmnet(X_lasso, Y_lasso,
                      family = "cox",
                      alpha = 1,
                      nfolds = 10,
                      type.measure = "C")
lambda_min <- lasso_cv$lambda.min
lambda_1se <- lasso_cv$lambda.1se
lasso_model <- glmnet(X_lasso, Y_lasso,
                      family = "cox",
                      alpha = 1,
                      lambda = lambda_1se)
lasso_coef <- coef(lasso_model)
selected_vars <- rownames(lasso_coef)[which(lasso_coef != 0)]
```

```
selected_vars <- selected_vars[selected_vars != "(Intercept)"]
cat("Variables selected by LASSO (", length(selected_vars), "):\n")
print(selected_vars)
create_lasso_coef_plot <- function(lasso_cv) {
lasso_full <- glmnet(X_lasso, Y_lasso, family = "cox", alpha = 1)
coef_data <- as.matrix(lasso_full$beta)
  lambda_values <- lasso_full$lambda
coef_long <- melt(coef_data)
  colnames(coef_long) <- c("Variable", "LambdaIndex", "Coefficient")
  coef_long$Lambda <- lambda_values[coef_long$LambdaIndex]
  coef_long$LogLambda <- log(coef_long$Lambda)
 non_zero_vars <- unique(coef_long$Variable[abs(coef_long$Coefficient) > 0])
  coef_long$Color <- "Other"
  coef_long$Color[coef_long$Variable == "EASIX"] <- "EASIX"
  coef_long$LineWidth <- ifelse(coef_long$Variable == "EASIX", 1.5, 0.8)

 p <- ggplot(coef_long, aes(x = LogLambda, y = Coefficient, group = Variabl
e)) +
    geom_line(aes(color = Color, size = LineWidth, alpha = Color)) +
    scale_color_manual(values = c("EASIX" = "#E69F00", "Other" = "gray70"))
 +
    scale_size_identity() +
    scale_alpha_manual(values = c("EASIX" = 1, "Other" = 0.6)) +
    geom_vline(xintercept = log(lambda_1se), linetype = "dashed", color = "red
") +
    geom_vline(xintercept = log(lambda_min), linetype = "dashed", color = "bl
ue") +
    labs(
      title = "LASSO Coefficient Paths",
      x = expression(log(lambda)),
      y = "Coefficient",
      color = "Variable"
    ) +
    theme_minimal() +
    theme(
```

```
      plot.title = element_text(hjust = 0.5, face = "bold", size = 14),
      axis.title = element_text(size = 12),
      legend.position = "none",
      panel.grid.major = element_line(color = "gray90"),
      panel.grid.minor = element_blank()
    ) +
    annotate("text", x = log(lambda_1se), y = max(coef_long$Coefficient) * 0.
9,
             label = "lambda.1se", color = "red", hjust = -0.1) +
    annotate("text", x = log(lambda_min), y = max(coef_long$Coefficient) * 0.
9,
             label = "lambda.min", color = "blue", hjust = -0.1)

  return(p)}
lasso_coef_plot <- create_lasso_coef_plot(lasso_cv)
lasso_cv_plot <- create_lasso_cv_plot(lasso_cv)
figure_s2 <- lasso_coef_plot + lasso_cv_plot +
  plot_annotation(
    title = "Figure S2: Variable Selection Using LASSO Regression",
    subtitle = "A: LASSO coefficient paths; B: 10-fold cross-validation for la
mbda selection",
    caption = paste("Variables selected by LASSO:", length(selected_vars),
                    "\nUsing lambda.1se for sparser model"),
    theme = theme(plot.title = element_text(hjust = 0.5, face = "bold", size =
16),
                  plot.subtitle = element_text(hjust = 0.5, size = 12))
  )
ggsave("Figure_S2_LASSO_Selection.png", figure_s2,
       width = 16, height = 8, dpi = 300)
```

#The Importance of Features in Three Machine Learning Methods

```
#XGBoost
xgb_data <- feature_selection_data[, model3_vars]
xgb_data[] <- lapply(xgb_data, function(x) if(is.factor(x)) as.numeric(as.character
(x)) else x)
```

```
dtrain <- xgb.DMatrix(data = as.matrix(xgb_data),
                      label = feature_selection_data$event_icu)
xgb_params <- list(
  objective = "survival:cox",
  eval_metric = "cox-nloglik",
  max_depth = 4,
  eta = 0.1,
  subsample = 0.8,
  colsample_bytree = 0.8,
  min_child_weight = 1)
set.seed(123)
xgb_model <- xgb.train(params = xgb_params,
                       data = dtrain,
                       nrounds = 100,
                       verbose = 0)
xgb_importance <- xgb.importance(model = xgb_model,
                                 feature_names = colnames(xgb_data))
top_xgb <- xgb_importance %>%
  arrange(desc(Gain)) %>%
  head(20)
cat("Top 10 XGBoost features:\n")
print(head(top_xgb, 10))

#Gradient Boosting
gbm_data <- feature_selection_data
gbm_data$time <- gbm_data$time_icu
gbm_data$event <- gbm_data$event_icu
set.seed(123)
gbm_model <- gbm(Surv(time, event) ~ .,
                 data = gbm_data[, c(model3_vars, "time", "event")],
                 distribution = "coxph",
                 n.trees = 100,
                 interaction.depth = 3,
                 shrinkage = 0.1,
                 cv.folds = 5,
```

```
                        verbose = FALSE)
best_iter <- gbm.perf(gbm_model, method = "cv")
gbm_importance <- summary(gbm_model, n.trees = best_iter, plotit = FALSE)
top_gbm <- gbm_importance %>%
  arrange(desc(rel.inf)) %>%
  head(20)
cat("Top 10 Gradient Boosting features:\n")
print(head(top_gbm, 10))

#SVM
svm_data <- feature_selection_data
svm_X <- as.matrix(svm_data[, model3_vars])
svm_Y <- as.factor(svm_data$event_icu)
svm_ctrl <- rfeControl(functions = caretFuncs,
                       method = "cv",
                       number = 5,
                       verbose = FALSE)
set.seed(123)
svm_profile <- rfe(x = svm_X,
                   y = svm_Y,
                   sizes = c(5, 10, 15, 20),
                   rfeControl = svm_ctrl,
                   method = "svmRadial",
                   tuneLength = 5,
                   trControl = trainControl(method = "cv", number = 5))
svm_selected <- predictors(svm_profile)
svm_importance <- data.frame(
  Feature = model3_vars,
  Importance = ifelse(model3_vars %in% svm_selected, 1, 0)) %>%
  arrange(desc(Importance)) %>%
  head(20)
cat("SVM selected features:", length(svm_selected), "\n")
print(svm_selected)
create_importance_plot <- function(data, method_name, color, top_n = 15) {plot
_data <- data %>%
```

```
    arrange(desc(data[, 2])) %>%
    head(top_n)
  if (method_name == "XGBoost") {
    colnames(plot_data)[1:2] <- c("Feature", "Importance")
  } else if (method_name == "Gradient Boosting") {
    colnames(plot_data)[1:2] <- c("Feature", "Importance")
  } else if (method_name == "SVM") {
    colnames(plot_data)[1:2] <- c("Feature", "Importance")
  }
 plot_data$Feature_Label <- plot_data$Feature
  for (i in 1:nrow(plot_data)) {
    if (plot_data$Feature[i] %in% names(variable_labels)) {
      plot_data$Feature_Label[i] <- variable_labels[[plot_data$Feature[i]]]
    }
  }
p <- ggplot(plot_data, aes(x = reorder(Feature_Label, Importance), y = Importan
ce)) +
    geom_bar(stat = "identity", fill = color, alpha = 0.8) +
    coord_flip() +
    labs(
      title = paste(method_name, "Feature Importance"),
      x = "",
      y = ifelse(method_name == "SVM", "Selected (1) / Not Selected (0)", "
Importance Score")
    ) +
    theme_minimal() +
    theme(
      plot.title = element_text(hjust = 0.5, face = "bold", size = 12),
      axis.text.y = element_text(size = 9),
      axis.text.x = element_text(size = 9),
      axis.title.x = element_text(size = 10),
      panel.grid.major = element_line(color = "gray90"),
      panel.grid.minor = element_blank()
    )
```

```
xgb_plot <- create_importance_plot(top_xgb, "XGBoost", color = "#E69F00", to
p_n = 15)
gbm_plot <- create_importance_plot(top_gbm, "Gradient Boosting", color = "#56
B4E9", top_n = 15)
svm_plot <- create_importance_plot(svm_importance, "SVM", color = "#009E73",
 top_n = 15)
figure <- (xgb_plot | gbm_plot | svm_plot) +
  plot_annotation(
    title = "Figure: Feature Importance from Three Machine Learning Methods
",
    subtitle = "A: XGBoost; B: Gradient Boosting; C: SVM",
    theme = theme(plot.title = element_text(hjust = 0.5, face = "bold", size =
16),
                  plot.subtitle = element_text(hjust = 0.5, size = 12))

#Summary of Feature Selection Results
feature_selection_summary <- data.frame(
  Method = c("LASSO", "XGBoost", "Gradient Boosting", "SVM"),
  Number_Selected = c(
    length(selected_vars),
    nrow(xgb_importance[xgb_importance$Gain > 0, ]),
    nrow(gbm_importance[gbm_importance$rel.inf > 0, ]),
    length(svm_selected)
  ),
  Top_Variable = c(
    ifelse(length(selected_vars) > 0, selected_vars[1], "None"),
    ifelse(nrow(top_xgb) > 0, as.character(top_xgb$Feature[1]), "None"),
    ifelse(nrow(top_gbm) > 0, as.character(top_gbm$var[1]), "None"),
    ifelse(length(svm_selected) > 0, svm_selected[1], "None")
  ),
  EASIX_Rank = c(
    ifelse("EASIX" %in% selected_vars, which(selected_vars == "EASIX"), N
A),
    ifelse("EASIX" %in% xgb_importance$Feature,
```

```r
                which(xgb_importance$Feature[order(-xgb_importance$Gain)] == "E
ASIX"), NA),
        ifelse("EASIX" %in% gbm_importance$var,
                which(gbm_importance$var[order(-gbm_importance$rel.inf)] == "EA
SIX"), NA),
        ifelse("EASIX" %in% svm_selected, which(svm_selected == "EASIX"), N
A)
    ),
    EASIX_Selected = c(
        "EASIX" %in% selected_vars,
        "EASIX" %in% xgb_importance$Feature,
        "EASIX" %in% gbm_importance$var,
        "EASIX" %in% svm_selected
    ))
write.csv(feature_selection_summary, "Feature_Selection_Summary.csv", row.name
s = FALSE)
```

#Build the final Cox model

```r
final_vars <- c("EASIX", "age", "weight", "sapsii", "apsiii", "apacheii")
final_vars <- final_vars[final_vars %in% colnames(train_data)]

cat("\nFinal selected variables for modeling:\n")
print(final_vars)
train_final <- train_data %>%
    select(all_of(final_vars), time_icu, event_icu) %>%
    na.omit()
final_cox <- coxph(Surv(time_icu, event_icu) ~ .,
                        data = train_final)

cat("\nFinal Cox model summary:\n")
print(summary(final_cox))
```

#Evaluate the model on the test set

```r
test_final <- test_data %>%
    select(all_of(final_vars), time_icu, event_icu) %>%
```

```
  na.omit()
test_predictions <- predict(final_cox,
                            newdata = test_final[, final_vars],
                            type = "risk")
roc_test <- roc(test_final$event_icu ~ test_predictions)
auc_test <- auc(roc_test)

cat("\nTest set performance:\n")
cat("AUC:", round(auc_test, 3), "\n")
pdf("ROC_Curves_Test_Set.pdf", width = 10, height = 8)
par(mfrow = c(1, 2))
plot(roc_test, main = paste("ROC Curve (AUC =", round(auc_test, 3), ")"),
     col = "blue", lwd = 2)
abline(a = 0, b = 1, lty = 2, col = "red")
dev.off()
```

#Nomogram

```
nomogram_data <- train_data %>%
  select(subject_id, all_of(final_vars), time_icu, event_icu) %>%
  na.omit()

ddist <- datadist(nomogram_data)
options(datadist = 'ddist')
cph_model <- cph(Surv(time_icu, event_icu) ~ .,
                 data = nomogram_data[, c(final_vars, "time_icu", "event_icu
")],
                 x = TRUE, y = TRUE, surv = TRUE, time.inc = 28)
nomogram <- nomogram(cph_model,
                     fun = function(x) 1 - exp(-exp(x)),
                     fun.at = c(0.05, 0.1, 0.2, 0.3, 0.4, 0.5, 0.6, 0.7, 0.8, 0.
9),
                     funlabel = "Risk of 28-day ICU Death",
                     lp = FALSE)

png("Nomogram_Final_Model.png", width = 1200, height = 800, res = 150)
plot(nomogram,
```

```
      col_grid = gray(c(0.8, 0.95)),
      cex.axis = 0.8,
      cex.var = 0.9,
      lmgp = 0.3)
dev.off()
```

#Model comparison (with traditional scoring system)

```
model_comparison <- data.frame(
  Model = c("Final Cox Model", sofa", "apsiii", "sirs", "sapsii", "oasis", "charls
on", "apacheii"),
  AUC = c(
    auc_test,
    auc(roc(test_final$event_icu, test_data$sofa[match(test_final$subject_id, test_d
ata$subject_id)])),
    auc(roc(test_final$event_icu, test_data$apacheii[match(test_final$subject_id, te
st_data$subject_id)])),
    auc(roc(test_final$event_icu, test_data$sapsii[match(test_final$subject_id, test_
data$subject_id)]))
  ))

cat("\nModel comparison (AUC):\n")
print(model_comparison)
model_comparison_plot <- ggplot(model_comparison, aes(x = reorder(Model, A
UC), y = AUC)) +
  geom_bar(stat = "identity", fill = "steelblue", alpha = 0.8) +
  geom_text(aes(label = sprintf("%.3f", AUC)), hjust = -0.2, size = 4) +
  coord_flip() +
  labs(
    title = "Model Performance Comparison (AUC)",
    x = "Model",
    y = "Area Under Curve (AUC)"
  ) +
  ylim(0, 1) +
  theme_minimal() +
  theme(plot.title = element_text(hjust = 0.5, face = "bold"))
```

```
ggsave("Model_Comparison.png", plot = model_comparison_plot, width = 10, he
ight = 6, dpi = 300)
```

#Hypothesis Test of Proportional Risk

```
set.seed(123)
data <- read_excel("MIMIC.data.xlsx", sheet = "Sheet1")
data <- data %>%
mutate(
time_icu = pmin(icu_day, 28),
event_icu = death_within_icu_28days,
time_hosp = pmin(hosp_day, 28),
event_hosp = death_within_hosp_28days,
EASIX = (first_lactate_dehydrogenase_ld * first_creatinine) / first_platelet_count,
race = factor(race),
gc = factor(gc),
mi = factor(mi),
ventilation = factor(ventilation),
hld = factor(hld),
aki = factor(aki),
crrt = factor(crrt),
sirs = factor(sirs),
dm = factor(dm),
vp = factor(vp),
sa = factor(sa)
)
icu_model3_vars <- c(
"EASIX", "race", "first_temperaturef", "gc", "mi", "ventilation",
"first_white_blood_cells", "hld", "first_nbps", "first_ptt",
"first_rr", "first_glucose", "first_po2", "weight", "aki",
"crrt", "dm", "first_albumin", "first_calcium_total",
"first_magnesium", "first_rdw", "first_hr", "vp", "sapsii",
"first_ph", "first_calculated_total_co2", "first_bilirubin_total",
"first_urea_nitrogen", "charlson", "first_anion_gap",
"first_lactate", "age", "oasis", "sofa", "apacheii", "apsiii",
```

```
"first_asparate_aminotransferase_ast", "first_alanine_aminotransferase_alt"
)
hosp_model3_vars <- c(
"EASIX", "race", "first_temperaturef",
"first_white_blood_cells", "first_ptt", "ventilation", "first_nbps",
"first_glucose", "first_po2", "first_rr", "weight", "aki", "crrt",
"dm", "first_magnesium", "first_rdw", "vp", "first_albumin",
"first_hr", "sa", "first_ph", "first_bilirubin_total",
"first_calculated_total_co2", "first_urea_nitrogen", "charlson",
"first_calcium_total", "first_anion_gap", "first_lactate", "age",
"oasis", "sofa", "apacheii", "apsiii", "sapsii"
)
icu_analysis_data <- data %>%
select(all_of(icu_model3_vars), time_icu, event_icu) %>%
na.omit()
hosp_analysis_data <- data %>%
select(all_of(hosp_model3_vars), time_hosp, event_hosp) %>%
na.omit()
icu_formula <- as.formula(paste("Surv(time_icu, event_icu) ~",
paste(icu_model3_vars, collapse = " + ")))
hosp_formula <- as.formula(paste("Surv(time_hosp, event_hosp) ~",
paste(hosp_model3_vars, collapse = " + ")))
icu_cox_model <- coxph(icu_formula, data = icu_analysis_data)
hosp_cox_model <- coxph(hosp_formula, data = hosp_analysis_data)
icu_ph_test <- cox.zph(icu_cox_model)
hosp_ph_test <- cox.zph(hosp_cox_model)
extract_ph_test_results <- function(ph_test, model_name) {
global_p <- ph_test$table["GLOBAL", "p"]
var_results <- data.frame(
Variable_Name = rownames(ph_test$table)[-nrow(ph_test$table)],
df = NA,
P_value = ph_test$table[-nrow(ph_test$table), "p"],
stringsAsFactors = FALSE
)
var_results$df <- 1
```

```
global_row <- data.frame(
Variable_Name = "Global",
df = nrow(ph_test$table) - 1,
P_value = global_p,
stringsAsFactors = FALSE
)
all_results <- rbind(var_results, global_row)
all_results$Model <- model_name
return(all_results)
}
icu_results <- extract_ph_test_results(icu_ph_test, "ICU Mortality (Model 3)")
hosp_results <- extract_ph_test_results(hosp_ph_test, "Hospital Mortality (Model 3)")
table_s8 <- icu_results %>%
select(Variable_Name, df, P_value) %>%
mutate(P_value = ifelse(P_value < 0.001, "<0.001",
ifelse(P_value < 0.01, format.pval(P_value, digits = 1),
format.pval(P_value, digits = 2)))) %>%
rename("Variable Name" = Variable_Name, "P-value" = P_value)
cat("Table S8: Multi-factor Cox regression (Model 3) proportional hazards
assessment\n")
print(table_s8, row.names = FALSE)
write.csv(table_s8, "Table_S8_Proportional_Hazards_Test.csv", row.names =
FALSE)
icu_ph_plot <- plot(icu_ph_test)
hosp_ph_plot <- plot(hosp_ph_test)
icu_easix_test <- cox.zph(icu_cox_model, terms = FALSE)
hosp_easix_test <- cox.zph(hosp_cox_model, terms = FALSE)
icu_easix_p <- icu_easix_test$table["EASIX", "p"]
hosp_easix_p <- hosp_easix_test$table["EASIX", "p"]
cat("\nEASIX-specific proportional hazards tests:\n")
cat("ICU Mortality Model - EASIX P-value:", format.pval(icu_easix_p, digits = 3),
"\n")
cat("Hospital Mortality Model - EASIX P-value:", format.pval(hosp_easix_p, digits =
3), "\n")
save(icu_ph_test, hosp_ph_test, table_s8,
```

icu_easix_p, hosp_easix_p,
